# Low molecular weight fucoidan inhibits hepatocarcinogenesis and nonalcoholic fatty liver disease in zebrafish via ASGR/STAT3/HNF4A signaling

**DOI:** 10.1002/ctm2.252

**Published:** 2017-07-28

**Authors:** Szu‐Yuan Wu, Wan‐Yu Yang, Chun‐Chia Cheng, Kuan‐Hao Lin, Bonifasius Putera Sampurna, Suat‐Ming Chan, Chiou‐Hwa Yuh

**Affiliations:** ^1^ Department of Food Nutrition and Health Biotechnology, College of Medical and Health Science Asia University Taichung 41354 Taiwan; ^2^ Big Data Center, Lo‐Hsu Medical Foundation Lotung Poh‐Ai Hospital Yilan 26546 Taiwan; ^3^ Division of Radiation Oncology, Lo‐Hsu Medical Foundation Lotung Poh‐Ai Hospital Yilan 26546 Taiwan; ^4^ Department of Healthcare Administration, College of Medical and Health Science Asia University Taichung Taiwan; ^5^ Graduate Institute of Business Administration Fu Jen Catholic University Taipei 24205 Taiwan; ^6^ Institute of Molecular and Genomic Medicine National Health Research Institutes Zhunan 35053 Taiwan; ^7^ Radiation Biology Research Center, Institute for Radiological Research Chang Gung University/Chang Gung Memorial Hospital at Linkou Taoyuan 33302 Taiwan; ^8^ Institute of Bioinformatics and Structural Biology National Tsing‐Hua University Hsinchu 30013 Taiwan; ^9^ Department of Biological Science & Technology National Chiao Tung University Hsinchu 30010 Taiwan; ^10^ PhD Program in Environmental and Occupational Medicine Kaohsiung Medical University Kaohsiung 80708 Taiwan

**Keywords:** hepatocellular carcinoma, oligo‐fucoidan, therapeutics, zebrafish

## Abstract

**Background:**

Hepatocellular carcinoma ranks fourth in cancer‐related mortality currently lacks effective therapeutics. Fucoidan is sulfated polysaccharide that is mainly found in brown seaweeds. In this study, we investigated the effects and mechanisms of low molecular weight fucoidan (i.e. oligo‐fucoidan [OF]) preventing hepatocarcinogenesis.

**Methods:**

We used [*HBx,src*], [*HBx,src,p53^−/+^*], and [*CD36*] transgenic zebrafish liver cancer model treated with OF, and performed molecular and histopathological analysis. Transcriptomic and pathways analysis was performed.

**Results:**

Decreased expression of lipogenic enzymes, fibrosis markers, and cell cycle/proliferation markers by OF in [*HBx,src*] and [*HBx,src,p53^−/+^*] transgenic fish. Liver fibrosis was decreased as revealed by Sirius Red staining, and the liver cancer formation was eventually reduced by feeding OF. OF was also found to be capable of reducing lipid accumulation and cancer formation in non‐B non‐C Hepatocellular carcinoma (HCC) model in CD36 transgenic zebrafish. Whole‐genome expression analysis showed that 661 genes were up‐regulated, and 451 genes were downregulated by feeding OF. Upregulated genes were mostly found in protein transporter activity, and downregulated genes were enriched with response to extracellular stimulus and metal binding in gene ontology analysis. The driver gene was *HNF4A* revealed by NetworkAnalyst from OF differential regulated genes at various insults. OF is able to bind the asialoglycoprotein receptor (ASGR) in hepatoma cells, and increased the phosphorylation of signal transducer and activator of transcription 3 (STAT3) in both hepatoma cells and [*HBx,src,p53^−/+^*] transgenic fish liver cancer model. Using chromatin‐immunoprecipitation, we found pSTAT3 could associate with the P1 promoter of HNF4A. Knockdown of either ASGR or HNF4A reversed OF mediated anti‐cancer cell proliferation.

**Conclusions:**

Taken together, we provide evidence that OF exhibits the anti‐HCC, anti‐steatosis, and anti‐fibrosis effect for liver in zebrafish models, and the anti‐cancer potential of OF attributed to the binding to ASGR and activation of STAT3/HNF4A signaling. OF might be potentially valuable for the management of HCC.

## Graphical abstract

- We used zebrafish preclinical model and whole‐genome transcriptome analysis to understand the mechanisms for oligo‐fucoidan anti‐HCC effect.
- Oligo‐fucoidan binds to ASGR receptor on hepatocyte.
- Oligo‐fucoidan activates the phosphorylation of STAT3, which binds to the P1 promoter of HNF4A, transcriptionally regulates P1‐isoform HNF4A expression.
- Oligo‐fucoidan prevents steatosis, fibrosis and HCC formation in HBx,src,p53_−_/^+^, diet induced obesity and CD36 transgenic fish.

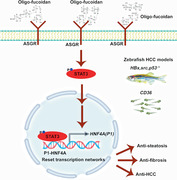

## 1 BACKGROUND

Liver cancer ranks fourth in the cancer‐related deaths; the burden is highest in East Asia and Africa.^1^, ^2^ Hepatocellular carcinoma (HCC), the most common type of liver cancer, comprises 75‐85% of liver cancer cases. The risk factors include chronic Hepatitis B virus (HBV) and Hepatitis C virus (HCV) infection, alcohol consumption, aflatoxin B1 exposure, and metabolic disorders.^3^ Due to the HBV vaccination and anti‐HCV drugs, the prevalence of viral associated HCC is decreased,^4^, ^5^, ^6^ however, non‐B non‐C (NBNC) HCC is increasing due to the prevalence of obesity.^7^, ^8^ The treatment for early to intermediate‐stage HCC includes resection, liver transplantation, ablation, transarterial therapies, and internal radiation therapy. For the advanced late stage HCC, systemic therapy is recommended; Sorafenib and Lenvatinib are the first‐line treatment; and Regorafenib, Cabozantinib, and Ramucirumab are the second‐line treatment.^9^ However, the first‐line can extend life for 11‐13 months, and second line treatment extend 8‐10 months, effective therapeutic means are urgently needed.^9^ Due to the heterogeneity of HCC, we need to have preclinical animal model for different risk factors of HCC and screen the anti‐HCC therapeutics.

The zebrafish (*Danio rerio*) is the third model organism in medical research, especially in the field of cancer research and drug screening.^10^, ^11^, ^12^ Zebrafish has become important model organism for liver cancer study recently.^10^ Previously, we found that aflatoxin B1 synergized with hepatitis B virus X‐antigen (HBx) can accelerate liver cancer formation.^13^ HBx synergized with *p53* mutation and *src* oncogene can cause more severe and early onset HCC.^13^ Moreover, diet‐induced obesity synergized with oncogenes leads to the higher incidence of HCC to zebrafish with the age of 5 months.^14^ Using the transgenic zebrafish HCC models, we can screen anti‐HCC therapeutics more efficiently.^15^ CD36 (cluster of differentiation 36), also known as fatty acid translocase, amplification occurs in NBNC HCC patients, and we created a CD36 transgenic fish. Thus, we used those zebrafish HCC models to investigate the anti‐HCC effect of low molecular weight fucoidan.

Fucoidan extracted from brown seaweeds is a type of sulfated polysaccharide that exhibits bioactivities for anti‐inflammatory, anti‐tumor effects, and fibrosis prevention.^16^, ^17^, ^18^, ^19^, ^20^ In hepatoma cell lines, fucoidan exhibits anti‐tumor effect through modulation of lncRNA expression,^21^ promotes apoptosis by via MAPK/ERK pathway,^22^ and increases the expression of microRNA‐29b to suppress DNMT3B‐MTSS1 axis.^23^ Fucoidan exhibits low affinity for bFGF,^24^ also can bind to fibronectin and inhibit the extracellular matrix‐receptor interaction,^25^ it can also bind to Toll‐like receptor 4 (TLR4) in lung cancer cells.^26^ The molecular structure of fucoidan is huge; thus it is believed that numerous unknown receptors to be discovered and determined.

Here, we examined the anti‐HCC effect of low molecular weight fucoidan, that is oligo‐fucoidan (OF) which has been demonstrated previously as a potential agonist for the C‐type lectin‐like receptor 2 in platelets.^27^ The major C‐type lectin‐like receptor 2 in hepatocyte is asialoglycoprotein receptor (ASGR).^28^ In addition, the sialic acid loss in platelets exposing galactose activates pSTAT3, where STAT3 is signal transducer and activator of transcription 3, by binding to ASGR in hepatocytes.^29^ Previously, we have demonstrated OF protects the liver through binding to ASGR and activates STAT3 in normal liver cells,^30^ and prevents radiation induced firbosis and secondary tumor.^31^ In this study, we investigated whether OF would be capable of reducing cancer incidence in different transgenic zebrafish HCC models including [*HBx,src,p53^−/+^*] transgenic zebrafish under diet induced obesity (DIO) developed HCC at 5 months of age,^14^ and CD36 transgenic zebrafish under high‐fat diet induced HCC at 1 month. Thus, we used those models to evaluate the efficacy of OF for anti‐HCC treatment for both HBV‐associate HCC and NBNC‐HCC.

## 2 MATERIALS AND METHODS

### 2.1 OF

The OF, manufactured by Hi‐Q Marine Biotech International Ltd. (Taipei, Taiwan), was extracted from seaweed, *Laminaria japonica*, with distilled water (100 g of dry seaweed/5 L) and boiled at 100°C for 30 minutes,^32^ and by adding glycolytic enzyme (1 mg of enzyme/g fucoidan for 6 hours) and passed through a 30 kDa molecular weight cut‐off membrane to obtain the low molecular weight fucoidan in a range between 500 and 1500 Da.^33^ The same material has been used in different experiments including in vitro and in vivo models, and demonstrated that OF exhibits hepatocyte protection and anti‐radiation induced fibrosis and secondary tumor.^30^, ^31^, ^34^

### 2.2 Zebrafish maintenance and transgenic zebrafish lines

The zebrafish (*Danio rerio*) [*HBx,src*] and [*HBx,src,p53^−/+^*] transgenic fish for this study were generated and bred in our laboratory. Tg(*fabp10a*:*HBx‐mCherry*, *myl7:EGFP*) and Tg(*fabp10a:src, myl7:EGFP*) were used to generate the double transgenic fish denoted as [*HBx,src*]; Tg(*fabp10a:HBx‐mCherry, myl7:EGFP*) × *tp53^zdf1/+^* and Tg(*fabp10a:src, myl7:EGFP*) were used to generate the triple transgenic fish denoted as [*HBx,src,p53^−/+^*]. All of the fishes were maintained in the Taiwan Zebrafish Core Facility (TZCF) at National Health Research Institute (NHRI) which has been AAALAC accredited since 2015. The animal protocol was approved by the Institution Animal Care and Use Committee (IACUC) of the NHRI (IACUC‐106118‐A).

### 2.3 Generation of CD36 transgenic fish

We generated the liver‐specific CD36 expression construct using Gateway cloning as described previously.^13^ The CD36 cDNAs were amplified using attB1‐CD36 and attB2‐CD36 primer from human cDNA library. Here is the sequence of attB1‐CD36 primer with ATG in the bold: GGGGACAAGTTTGTACAAAAAAGCAGGCT**ATG**GGCTGTGACCGGAAC.

The sequence of attB2‐CD36 primer is following with the terminal codon in the bold: GGGGACCACTTTGTACAAGAAAGCTGGGT**TTA**TTTTATTGTTTTCGATCTGCATG.

BP reaction (a recombination reaction between the attB sites of the PCR products and the attP sites on the donor vector) was used to generate the pME‐CD36 (middle entry clone) with pDONR221 vector, 50 fmol of cleanup CD36 polymerase chain reaction (PCR) product, and BP clonase. After sequencing confirmation the pME‐CD36 contained correct sequence, LR reaction (a recombination reaction between the attL sites of the entry clones and the attR sites on the destination vector) was used to generate the final expression construct with pDEST‐Tol2‐ cmlc2:GFP vector, p5E‐fabp10a, pME‐CD36, p3E‐pA, and LR clonase enzyme. After sequencing confirmation the pTol2‐fabp10a:CD36‐ cmlc2:GFP contained correct sequence, the expression construct was injected together with Tol2‐transposase mRNA to AB(WT) zebrafish embryos, and created the Tg(fabp10a:CD36:cmlc2:GFP) transgenic fish according to the standard protocol.

### 2.4 Dietary procedure, OF administration, and tissue collection for quantitative polymerase chain reaction and hematoxylin and eosin stain

The [*HBx,src*] and [*HBx,src,p53^−/+^*] transgenic zebrafish were allocated to two different diets: normal diet (NOR) with 6.9 mg cysts/fish/day of Artemia and diet‐induced obesity (DIO) with 83 mg cysts/fish/day of Artemia for 2 months starting at 3 months of age as described previously.^14^ OF (0.051 mg in 5 μL) was administrated by oral gavage thrice a week for 1 month starting at 4 months of age, by injected slowly into the intestinal tract of anesthetized (with 150 mg/L MS‐222) adult fish using flexible tubing as described previously.^31^ After 1 month of OF oral feeding, we sacrificed the fish, extracted the RNA, and paraffin embedding for H&E stain was performed as described previously.^30^, ^31^ The quantitative real‐time PCR (qPCR) primers are listed in Table 1.

**TABLE 1:** The primer information for qPCR analysis in human cell line and zebrafish

Gene name	Primer name	Sequence (5′ to 3′)	Accession number	Size (bp)
Lipogenic factor				
*pparg*	Q‐pparg ‐F	GGTTTCATTACGGCGTTCAC	NM_131467.1	250
	Q‐pparg ‐R	TGGTTCACGTCACTGGAGAA		
*srebf1*	Q‐srebf1‐F	CATCCACATGGCTCTGAGTG	NM_001105129.1	250
	Q‐srebf1‐R	CTCATCCACAAAGAAGCGGT		
*Mlxip*	Q‐mlxip ‐F	GGAGATGGACTCGCTCTTTG	XM_001338467	200
	Q mlxip‐R	GCAGAGGCTCAAAAGTGTCC		
Lipogenic enzyme				
*agpat4*	Q‐agpat4‐F	TTGGCGAAAAAGGAACTGTC	NM_212992	250
	Q‐agpat4‐R	GGTGGTACTTGAGTTTGGGG		
*pap*	Q‐pap‐F	CAGTTCTTCCTGATTGCTGC	XM_692415	250
	Q‐pap‐R	TCCTCAAAGCTTAGTTCGGG		
*fasn*	Q‐fasn‐F	ATCTGTTCCTGTTCGATGGC	XM_682295	250
	Q‐fasn‐R	AGCATATCTCGGCTGACGTT		
Fibrosis marker genes				
*ctgfa*	Q‐ctgfa‐F	TGTGTGTTTGGTGGAATGGT	NM_001015041.2	198
	Q‐ctgfa‐R	GGAGTCACACACCCACTCCT		
*hpse*	Q‐hpse‐F	GCTCTGGTTTGGAGCTCATC	NM_001045005.1	203
	Q‐hpse‐R	GAAATCCCGACCAAGTTGAA		
*col1a1a*	Q‐col1a1a‐F	TATTGGTGGTCAGCGTGGTA	NM_199214.1	199
	Q‐col1a1a‐R	TCCTGGAGTACCCTCACGAC		
Cell cycle/proliferation‐related genes				
*ccne1*	Q‐ccne1‐F	TCCCGACACAGGTTACACAA	NM_130995.1	201
	Q‐ccne1‐R	TTGTCTTTTCCGAGCAGGTT		
*cdk1*	Q‐cdk1‐F	CTCTGGGGACCCCTAACAAT	NM_212564.2	200
	Q‐cdk1‐R	CGGATGTGTCATTGCTTGTC		
*cdk2*	Q‐cdk2‐F	CAGCTCTTCCGGATATTTCG	NM_213406.1	199
	Q‐cdk2‐R	CCGAGATCCTCTTGTTTGGA		
Primers for validate the genes from microarray				
*hnf4a*	Q‐13279312‐F	AGCCGTGTGGCTGTAAGAAT	NM_194368.1	250
	Q‐13279312‐R	GTAGTGTCGGCAACAGCAGA		
*arl5c*	z‐arl5c F	CTCCTGTTCGCCAAATTGAT	NM_200846.1	212
	z‐arl5c R	AGACTCTCCTGCCCTCCAAT		
*gadd45ba*	z‐gadd45ba F	TCACAGTCGGCGTTTATGAG	NM_213031.3	155
	z‐gadd45ba R	GATGTCGTTATCGCAGCAGA		
*foxo3b*	z‐foxo3b F	AGAGAGCACCCCTGACAAGA	NM_131085.1	189
	z‐foxo3b R	CACGAGCTCTTTCCAGTTCC		
*slc19a2*	z‐slc19a2 F	CATGGCTTTACCCGACAGTT	XM_005168735.4	198
	z‐slc19a2 R	GGTAATCTGTGGCCAGGAAA		
*pck1*	z‐pck1 F	GAAACTCACTGCTGGGGAAG	NM_214751.1	221
	z‐pck1 R	GTCTCCCACACACTCCACCT		
*rorab*	z‐rorab F	AGGATGACAAAACCGGTGAC	NM_201067.1	206
	z‐rorab R	GGGCTGAATGTCCAGGTAAA		
*mxd3*	z‐mxd3 F	GTTATGCCTCCGTTCTTCCA	NM_201056.1	213
	z‐mxd3 R	GCAGGTTCAGGGTTGTGTTT		
*cpb1*	z‐cpb1 F	CAGAGCGACATGGAAGTCAA	NM_001328425.1	213
	z‐cpb1 R	GTGCATGGTTCTTCCCTCAT		
*gtf3c5*	z‐gtf3c5 F	GCGGTCTCATCAGACACAGA	XM_688483.8	219
	z‐gtf3c5 R	GCGGAGCTCAATTCTTTTTG		
*hnf4a(P1)*	hnf4a_P1_F	GATGGCAGACTATAGCGAGGC	ENSDARG00000021494.11	239
	hnf4a_P2/P1_R	TTTTGCGTACACTGCGTCTG		
*hnf4a(P2)*	hnf4a_P2_F	TAGCACCCATATGGAGGCACC	ENSDARG00000021494.11	182
	hnf4a_P2/P1_R	TTTTGCGTACACTGCGTCTG		
*ddah1*	Q‐13273615‐F	GATCCTGGCCAACACCTTTA	NM_213276.1	229
	Q‐13273615‐R	CGGCAGGTTCATGTACACAC		
*11b‐hsd1*	Q‐13092949‐F	TTGCTGATTGCTGTCCTCAC	XM_009298924.3	216
	Q‐13092949‐R	CTTAGCGCCCAGTTTCTCAC		
*zhi(asgr1)*	zhi(asgr1)‐F	TGGAAAACTGCAGAAAGCAA	XM_005167373.3	242
	zhi(asgr1)‐R	CAATCCTCACCATTCACACG		
*tdo2a*	tdo2a‐F	GTTCCTTTCCAGCTGCTGAC	NM_001102616.2	195
	tdo2a‐R	CGTGGCCAGGTTAAACAGAT		
*actin*	Q‐actin ‐F	CTCCATCATGAAGTGCGACGT	NM_131031.1	180
	Q‐actin ‐R	CAGACGGAGTATTTGCGCTCA		
HNF4A promoter (ChIP qPCR)				
*P1‐HNF4A*	h‐HNF4A‐promoter P1 F	GACGGTAGGTGCCTGAATGT	AH005099.2	225
	h‐HNF4A‐promoter P1 R	GGAGCAGAATGGACTGGAAG		
*P2‐HNF4A*	h‐HNF4A‐promoter P2 F	CAGCATCCAGTAGGCACTCA	NG_009818.1	216
	h‐HNF4A‐promoter P2 R	AACCCAGAGCCAGGTGTATG		
Primers for CD36 larva				
*ccne1*	Q‐ccne1‐F	TCCCGACACAGGTTACACAA	NM_130995.1	201
	Q‐ccne1‐R	TTGTCTTTTCCGAGCAGGTT		
*srebf1*	Q‐srebf1‐F	CATCCACATGGCTCTGAGTG	NM_001105129.1	250
	Q‐srebf1‐R	CTCATCCACAAAGAAGCGGT		
*hmgcs1*	Q‐hmgcs1‐F	CTCACTCGTGTGGACGAGAA	NM_201085.2	141
	Q‐hmgcs1‐R	GATACGGGGCATCTTCTTGA		
*acox3*	Q‐acox3‐F	AAGGACATCGAGCGAATGAT	NM_213147.1	250
	Q‐acox3‐R	CTATGAAAGAGTGGAGGCCG		
*atf6*	Q‐atf6‐F	CTGTGGTGAAACCTCCACCT	NM_001110519.1	200
	Q‐atf6‐R	CATGGTGACCACAGGAGATG		
*il6*	Q‐il6‐F	AGACCGCTGCCTGTCTAAAA	NM_001261449.1	136
	Q‐il6‐R	TTTGATGTCGTTCACCAGGA		
*eef1a1*	Q‐eef1a1‐F	TACTTCTCAGGCTGACTGTG	NM_131263.1	228
	Q‐eef1a1‐R	ATCTTCTTGATGTATGCGCT		

The CD36 zebrafish larvae were fed four times daily starting at 5‐day post‐fertilization (dpf) until 15 dpf or 30 dpf, with NOR containing 12% fat or high fat diet (HFD) with 24% fat,^35^ supplement with larvae with 20 mL Paramecium in 800 mL water, and the zebrafish were immersed in 8 mg/mL OF solution every night (from 5 pm to 9 am) in a petri dish.

### 2.5 Examination of lipid accumulation in CD36 transgenic zebrafish larva

Zebrafish feeding started at 5 dpf according to the diet treatment for each group until 15 dpf, and then fasting for 2 days after the final feeding time. Food and drug treatments were stopped at this point to minimize the false positive background.

For whole body Oil Red O staining, a batch of 17 dpf zebrafish was euthanized using high concentration of tricaine. Zebrafish were fixed in the 4% paraformaldehyde (PFA) in PBS for overnight at 4°C. PFA was disposed and washed twice with phosphate buffered saline (PBS) for 5 minutes each. Larvae were then infiltrated with 80% and 100% propylene glycol at room temperature for 20 minutes, respectively. Fish were transferred into microcentrifuge tube (five fish each tube) filled with 0.5% Oil Red O (Sigma‐Aldrich Inc., St. Louis, MO) in 100% propylene glycol for overnight at room temperature in the dark place. The next day, fish were washed with PBS twice for 5 minutes each. Larvae were infiltrated with 100% and 80% propylene glycol separately in order at room temperature for 20 minutes. Picture of individual fish was taken by using bright field microscope. Larvae were pooled together in the microcentrifuge tube and washed with PBS. Four percent NP‐40 made up in the 100% isopropanol was added and incubated overnight at room temperature, avoid light exposure. On the final day, immersed solution was transferred into a 96‐well plate (triplicate). The absorbance was read at 490 nm and 570 nm as the reference.

For LipidGreen staining, another batch of the 17 dpf zebrafish was immersed in a 90 mm petri dish filled with 10 μM LipidGreen2 (Merck Inc., Kenilworth, NJ) solution made up in fish water and incubated at 28°C for 15 minutes. Larvae were transferred into a new dish containing fresh fish water. Anesthetization was done using tricaine (Sigma‐Aldrich Inc., St. Louis, MO), and fish were placed on the agar plate for imaging using green fluorescence. The liver area was selected, and fluorescence intensity was quantified using the ImageJ software.

The expression levels of lipogenesis gene: sterol regulatory element binding transcription factor 1(*srebf1*), cholesterol synthesis gene: 3‐Hydroxy‐3‐Methylglutaryl‐CoA Synthase 1 (*hmgsc1*), lipid oxidation gene: Acyl‐CoA oxidase 3 (*acox3*), inflammation gene: interleukin 6 (*il6*), ER stress genes: activating transcription factor (*atf6*), and cell cycle/proliferation marker: cyclin E1 (*ccne1*) were analyzed from pool of 20 larvae. The primers are listed in Table 1, and eukaryotic translation elongation factor 1 alpha 1 (*eef1a1*) was used as an internal control as described previously.^35^

### 2.6 Sirius Red stain

Fish hepatic tissue sections were stained by Sirius Red Stain (Polyscience, Warrington, PA) to evaluate liver fibrosis. Slides were deparaffinized and hydrated with deionized (DI) water, then stained with Wiegert's hematoxylin for 8 minutes and rinsed well in DI water. Slides were placed in solution A (phosphomolybdic acid) for 2 minutes and rinsed in DI water, then placed in solution B (Picrosirius Red F3BA stain) for 60 minutes, then placed in solution C (0.1 N hydrochloric acid) for 2 minutes, and then rinsed in 70% ethanol for 45 seconds. Slides were then dehydrated, cleared, and mounted. Images were captured by Leica DM2500 Microsystems equipped with color CCD (DP73, Olympus Inc., Tokyo, Japan).

### 2.7 Immunohistochemistry analysis

The immunohistochemistry (IHC) analysis was performed by following the previous methodology.^13^ Primary antibodies including proliferating cell nuclear antigen (PCNA) (Santa Cruz, CA) and pSTAT3 (GeneTex Inc., CA) were stained in hepatic tissues of zebrafish. Data were analyzed by immunoreactive scores (IRSs) which were calculated by intensity (score 1‐3) multiply proportion of positive cells (score 1‐4).^36^

### 2.8 Chromatin immunoprecipitation qPCR assay

To analyze the pSTAT3 binding ability, we added 5 μg of pSTAT3 antibody (Cell Signaling Inc., Danvers, MA) to perform chromatin immunoprecipitation (ChIP) (Merck). Two microliters of ChIP samples were used for qPCR analysis, as described previously,^37^ using gene‐specific primers (Table 1). For the ChIP data, we adjusted the signals to IgG control. To analyze the ChIP‐qPCR, the Ct minus the Ct from input control to obtain the ΔCT, and then the ΔCT minus the ΔCt from negative control IgG to obtain the ΔΔCT. The fold change was converted from ΔΔCT by 1.94ˆ(ΔΔCt).

### 2.9 GeneTitan array for gene expression profiling

The whole‐genome transcriptome analysis was performed and analyzed as described previously.^30^, ^31^

### 2.10 In vitro competition assay

HepG2 cells were used for in vitro competition assay as described previously.^30^

### 2.11 Positron Emission Tomography (PET) imaging for in vivo competition assay

The in vivo competition assay was performed by using adult Balb/c mice and the ^68^Ga‐NOTA‐hexavalent lactoside (HL) which was capable of binding to ASGR in the body,^38^ as described previously.^30^

For time‐dependent analysis of in vivo competition assay under the presence of OF, 100 mg OF was intravenously prescribed 1 hour prior to ^68^Ga‐NOTA‐HL injection. The PET imaging was acquired by Nano‐PET/CT Mediso Medical Imaging Systems (Mediso Inc., Budapest, Hungary) at 0, 2, and 4 hours after ^68^Ga‐NOTA‐HL injection.

### 2.12 Transfection by lentivirus system

293T cells were seeding at the density of 2 × 10^5^ cell/well in a 6‐well plate, after culture at 37℃ and 5% CO_2_ for overnight; lentivirus component (R8.91 and pMDG) and shRNA were added into cell medium. After culture for 2 days, until the cell confluence reached 70‐80%, collected the medium into micro‐centrifuge tube and spin‐down to separate cell from the supernatant, then moved 500 μl supernatant which containing lentivirus into Hep3B cell culture for overnight, and used 1 μg/ml puromycin as selection. Here is the shRNA sequence: shASGR1#1:CCGGGCACCACATAGGCCCTGTGAACTCGAGTTCACAGGGCCTATGTGGTGCTTTTTG; shASGR1#2:CCGGACGTGAAGCAGTTCGTGTCTGCTCGAGCAGACACGAACTGCTTCACGTTTTTTTG; shHNF4A#1:CCGGCCATCACCAAGCAGGAAGTTACTCGAGTAACTTCCTGCTTGGTGATGGTTTTT; shHNF4A#2:CCGGCGAGCAGATCCAGTTCATCAACTCGAGTTGATGAACTGGATCTGCTCGTTTTT.

### 2.13 Cell viability assay

5000 cells were cultured in a well of 96‐well plate with 100 μl culture medium, and treated with various concentrations of OF for 48 hours at 37°C and 5% CO_2_. For viability assay, 10 μl of WST‐1 reagent (BioVision Inc., Milpitas, CA) was added to each well and incubated at for 1 hour, then measured the absorbance of samples using a microtiter plate reader at 450 nm according to the filters available for the plater reader, and the reference wavelength should be 650 nm.

### 2.14 Western blot analysis

Western blot assay was performed as described previously^39^ with the specific antibodies listed below: Anti‐Asialoglycoprotein Receptor 1 antibody (ASGR1‐Ab, ab254262, Abcam Inc., Cambridge, MA), Phospho‐Stat3 (Tyr705)(D3A7) XP Rabbit mab (pSTAT3 Ab, #9145, Cell Signaling Inc., Danvers, MA), Anti‐h/m/rSTAT3 purified mouse monoclonal IgG (STAT3 Ab, #MAB1799, R&D Systems Inc., Minneapolis, MN), GAPDH antibody (GTX100118, GeneTex Inc., CA), Mouse IgG antibody (HRP) (GTX213111‐01, GeneTex Inc., CA), Rabbit IgG antibody (HRP) (GTX213110‐01, GeneTex Inc., CA).

### 2.15 Statistical analysis

We used unpaired Student's *t*‐test for analyzed the statistical significance of qPCR, and chi‐square test for analyzed the statistical significance of histopathological data. The statistical significance is shown as: * *P* ≤ .05, ** *P* ≤ .01, ***P ≤ .001, and ****P ≤ .0001.

## 3 RESULTS

### 3.1 OF pretreatment in adult HBx, src transgenic zebrafish decreased lipogenesis and fibrosis

**FIGURE 1:**
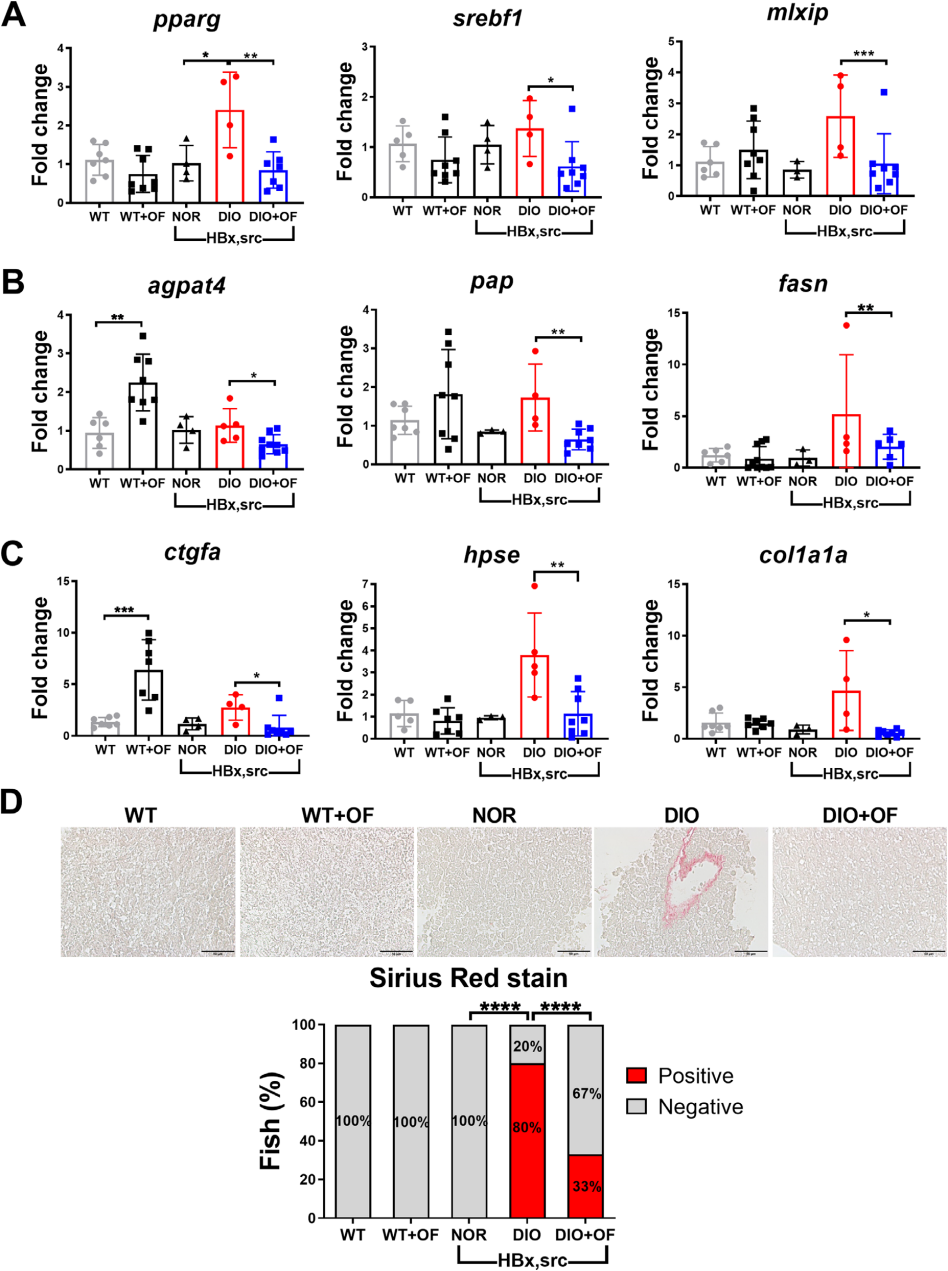
Oligo‐fucoidan pre‐treatment reduced the expression of lipogenesis genes, fibrosis marker and decreased the levels of collagen fibers in adult [*HBx,src*] transgenic zebrafish. **A**, Expression of lipogenic factors (*pparg*, *mlxip*, and *srebf1*), **B**, lipogenic enzymes (*agpat4*, *pap* and *fasn*), and **C**, fibrosis markers (*ctgfa*, *hpse*, and *col1a1a*). Data are presented as dot plots, each dots represent one fish. Statistical significance was calculated by *t*‐test (*P ≤ .05, **P ≤ .01, ***P ≤ .001). **D**, Representative images and statistical analysis of Sirius Red stain in [*HBx,src*] transgenic fish. Scale bar represents 50 μm. WT represents non‐transgenic wild‐type control fish, WT+OF stands for WT fish feeding with OF, NOR indicates normal diet, DIO denotes diet‐induced obesity, DIO+OF represents DIO fish given oral gavage with 0.051 mg OF.

Previously, we used the transgenic zebrafish model to demonstrate that liver‐specific overexpression of *HBx* and *src* oncogene promotes hepatocarcinogenesis, including steatosis and fibrosis in the earlier stages and hyperplasia, dysplasia, and HCC in the later stages,^13^ diet‐induced obesity can accelerate hepatocarcinogenesis.^14^ Therefore, to examine the anti‐HCC effect of OF, after 1 month oral‐feeding with OF to the [*HBx*,*src*] transgenic fish with diet‐induced obesity, we measured the expression of lipogenic factors, lipogenic enzyme, and fibrosis markers using qPCR. As control, there was no significant increase of lipogenic factors in non‐transgenic fish type oral feeding with OF (WT+OF) compared to WT (Figure 1A). However, the expression of lipogenic factors (peroxisome proliferator‐activated receptor‐gamma (*pparγ*), sterol regulatory element binding proteins‐1 (*srebf1*) and MLX interacting protein (*mlxip*)) was increased in diet‐induced obesity (DIO) compared to NOR in the [*HBx,src*] transgenic fish, and OF treatment significantly reduced the expression (Figure 1A). The expression of lipogenesis enzymes acyl‐coA:glycerol‐3‐phosphate acyltransferase 4 (*agpat4*) in WT+OF compared to WT was increased (Figure 1B), the pancreatitis‐associated protein (*pap*) and fatty acid synthase (*fasn*) were no difference in WT+OF compared to WT (Figure 1B). However, the lipogenesis enzyme was increased in DIO compared to NOR in the [*HBx,src*] transgenic fish, and reversed by OF treatment (Figure 1B). For the fibrosis makers (*ctgfa*, *hpse*, and *col1a1a*), there was a slightly increase of *ctgfa*, but not for *hpse* and *col1a1a* in WT+OF compared to WT (Figure 1C). The expression of fibrosis markers was elevated in [*HBx,src*] transgenic fish with DIO compared to NOR, OF significantly reduced the expression of fibrosis markers (Figure 1C). Reduction of collagen accumulation by OF was revealed by Sirius Red staining (Figure 1D). Taken together, these data suggest that OF treatment eradicated oncogene‐ and diet‐induced steatosis and hepatic fibrosis.

### 3.2 OF pretreatment in adult [HBx,src] and [HBx,src,p53^−/+^] transgenic zebrafish decreased HCC formation

**FIGURE 2:**
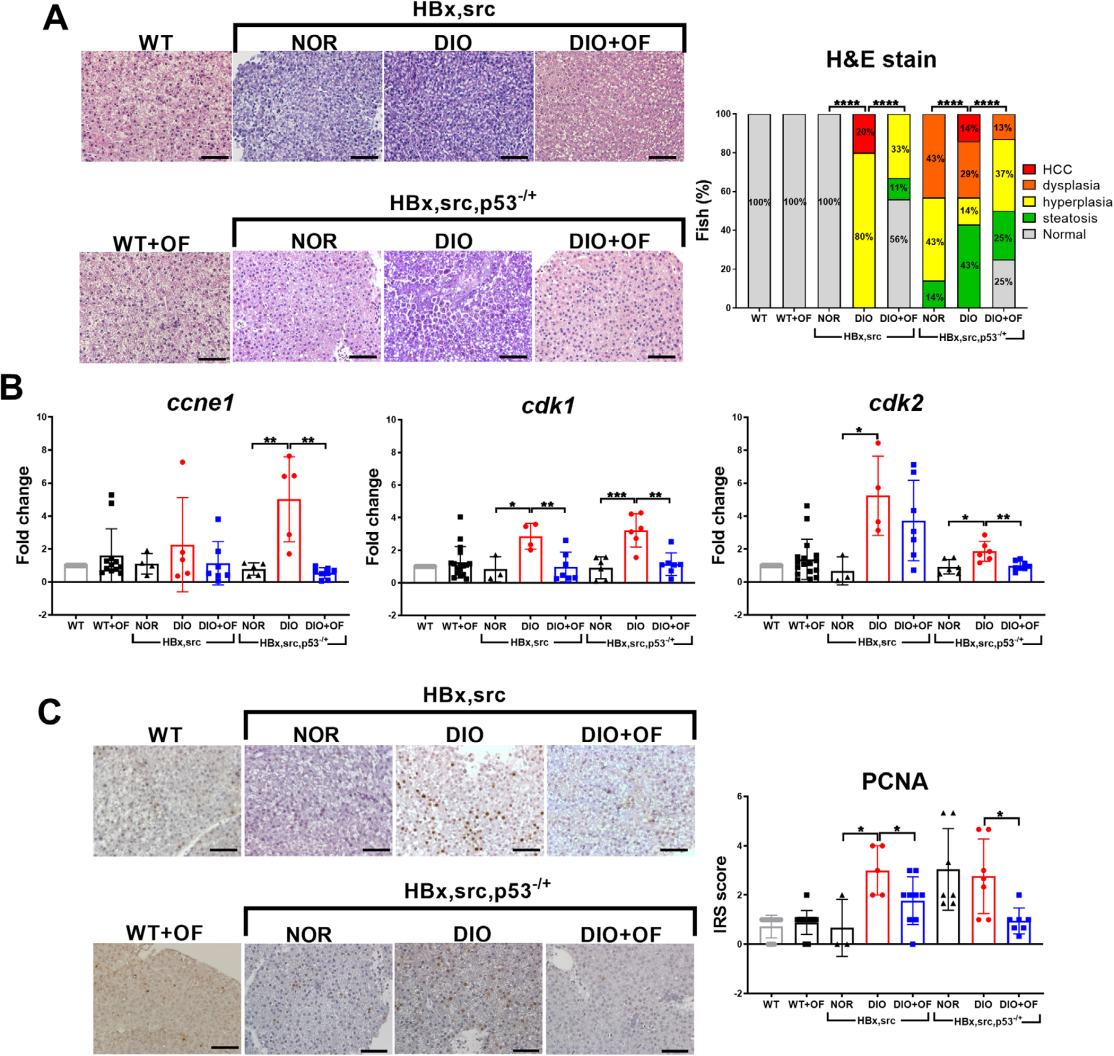
Oligo‐fucoidan pre‐treatment suppressed oncogene‐ and diet‐induced obesity‐mediated carcinogenesis in adult [*HBx,src*] and [*HBx,src,p53^−/+^*] transgenic zebrafish. A Representative H&E images show typical pathological tissue structures in hepatic tissues of [*HBx,src*] and [*HBx,src,p53^−/+^*] transgenic zebrafish that had been fed by normal diet (NOR, N > 4), diet‐induced obesity (DIO, N > 9), and DIO with OF (DIO+OF, N > 9). Both the non‐transgenic wild‐type control fish (WT) oral feeding with OF (WT+OF) show normal hepatocyte. The incidences of pathological alterations from two transgenic fish fed with distinct diets were shown as stacking bar chart. Statistical significance was calculated by chi‐square analysis (****P ≤ .0001). B, Analyses of mRNA levels of cell proliferation markers: *ccne1*, cyclin‐E1; *cdk1*, cyclin‐dependent kinase‐1; *cdk2*, cyclin‐dependent kinase 2 in [*HBx,src*] and [*HBx,src,p53^−/+^*] with normal diets (NOR), overfeeding (DIO), or DIO together with OF (DIO+OF), WT and WT+OF indicate non‐transgenic wild‐type fish without OF and with OF. Data are presented as dot plots. Statistical significance was calculated by *t*‐test (*P ≤ .05, **P ≤ .01, ***P ≤ .001). C, Representative proliferating cell nuclear antigen (PCNA) stain images in hepatic tissues of [*HBx,src*] and [*HBx,src,p53^−/+^*] transgenic zebrafish fed by different diets and OF. The corresponding immunoreactive scores (IRSs) of the PCNA staining were calculated by multiplying the staining intensity by the proportion of positive cells. Scale bars equal to 50 μm. Data are presented as dot plots. Statistical significance was calculated by *t*‐test (*P ≤ .05).

Next, we examined histopathological changes following OF treatment in transgenic HCC zebrafish using hematoxylin and eosin (H&E) staining. We classified the histopathological features, followed the criteria set by National Toxicology Program,^40^ and those guidelines were used in many literatures including ours.^13^, ^14^, ^31^, ^41^, ^42^, ^43^ The characteristic of hyperplasia was atypical hepatocytes with enlarged and mildly irregular nuclei as accelerated proliferation, dysplasia was defined by nuclear atypia, nuclear pleomorphism, and multinucleation, and HCC was characterized with more severe proliferation with enlargement of polymorphic nuclei, prominent nucleoli, and an increased number of mitotic figures. The lipid accumulates in the hepatocytes as vacuoles was defined as steatosis. The examples for HCC, dysplasia, hyperplasia, steatosis, and normal hepatocyte are shown in the Figure S1. As control, WT and WT+OF were all normal in H&E staining, [*HBx,src*] transgenic zebrafish overfed (DIO group) displayed hyperplasia (80%) and HCC (20%), in contrast to the zebrafish fed a NOR, which were 100% normal according to H&E staining (Figure 2A). Feeding the DIO fish with OF (DIO+OF) significantly decreased the incidence of hyperplasia from 80% to 33% and eradicated HCC, and the hepatocytes were normal or showed steatosis (Figure 2A). We also used other transgenic fish that overexpressed *HBx* and *src* in the liver against a *p53* mutant background (Figure 2B). With a NOR, [*HBx,src,p53^−/+^*] fish developed steatosis, hyperplasia, and dysplasia, and 2 months of overfeeding induced HCC formation in 5‐month‐old zebrafish as described previously.^14^ OF (DIO+OF) overfeeding for 1 month significantly decreased the incidence of various liver diseases (Figure 2A).These data indicate that OF effectively decreased the risk of oncogene‐ and overfeeding‐induced HCC.

Next, we examined the expression of cell cycle/proliferation markers such as cyclin E1 (*ccne1*), cyclin‐dependent kinase 1 (*cdk1*), and cyclin‐dependent kinase 2 (*cdk2*) (Figure 2B). There is a significant increase of *ccne1* for wild‐type treated OF (WT+OF), and also increase of *cdk1* and *cdk2* but not statistically significant. These results are consistent with previous finding that OF treatment increase the cell viability of normal cells.^30^ Pretreatment with OF (Figure 2B) diminished the expression of cell cycle/proliferation markers that were induced by DIO (Figure 2B), especially the expression of *cdk1* and *cdk2*. We also examined PCNA in liver specimens using IHC analysis staining. As control, WT and WT+OF exhibited very low PCNA staining, however, in [*HBx,src*] and [*HBx,src,p53^−/+^*] transgenic fish, the protein levels of PCNA were increased in the DIO fish, and it was eradicated by OF pretreatment (Figure 2C). These data suggest that OF treatment decreased the possibility of cancer formation induced by oncogenes and diet‐induced obesity.

### 3.3 OF exhibits anti‐lipid accumulation and anti‐HCC formation in CD36 transgenic fish

**FIGURE 3:**
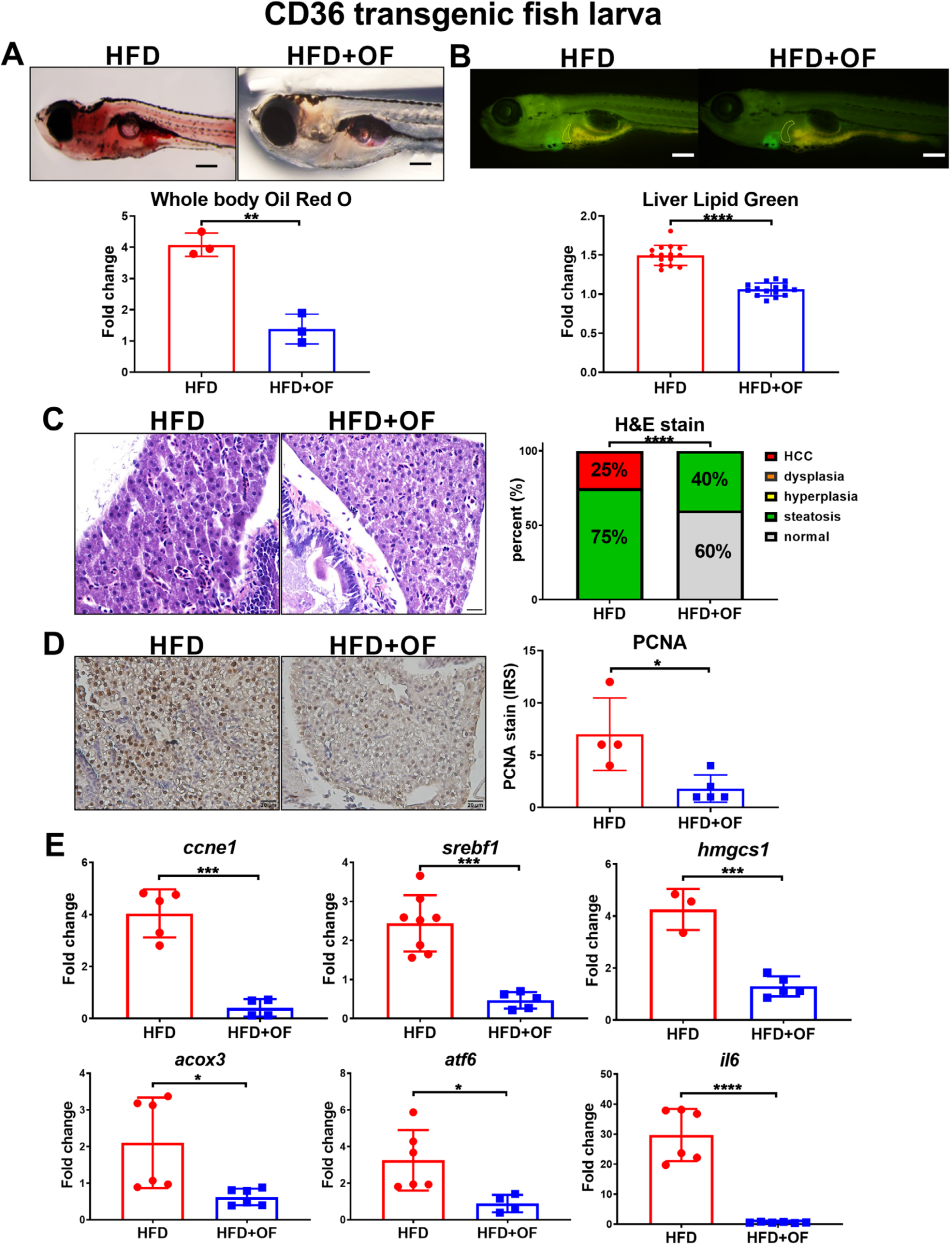
Oligo‐fucoidan exhibits anti‐lipid accumulation and anti‐HCC effect in *CD36* transgenic fish model. A, The anti‐lipid accumulation effect of fucoidan in a 15‐day‐old *CD36* transgenic fish model revealed by Oil Red staining from *CD36* transgenic fish fed with 24% high fat diet without or with fucoidan. The statistical analysis revealed significant reduction of Oil Red staining from OF treatment, each dot represent 10 larvae. B, The anti‐lipid accumulation effect of fucoidan in a 15‐day‐old *CD36* transgenic fish model revealed by LipidGreen staining from *CD36* transgenic fish fed with 24% high fat diet without or with fucoidan. The statistical analysis revealed significant reduction of lipid‐green staining from OF treatment, each dot represents 1 larva. C, The anti‐HCC effect of oligo‐fucoidan in a 1‐month‐old *CD36* transgenic fish model revealed by H&E staining. *CD36* transgenic fish fed with 24% high fat diet without and with fucoidan. Statistical analysis of H&E stain revealed significant reduction of steatosis and HCC formation upon OF treatment. D, Oligo‐fucoidan reduced the protein levels of PCNA proliferation marker in hepatic tissues of *CD36* transgenic zebrafish fed by high fat diet and OF. Statistical analysis of PCNA IHC stain revealed significant reduction of PCNA nuclear staining upon OF treatment. E, The gene expression profiles of the selected marker genes of lipogenesis, cholesterol synthesis, inflammation, ER stress, and cell proliferation of oligo‐fucoidan treatment in a 1‐month‐old *CD36* transgenic fish model. Statistical significance was calculated by *t*‐test: *P < .05, ***P ≤ .001; ****P ≤ .0001.

Non‐alcoholic fatty liver disease (NAFLD) is a major risk factor where the accumulation of fatty acids and lipids from de novo lipid synthesis in the liver gives rise to liver inflammation that progresses to HCC. *CD36*, fatty acid translocase, plays important role in NAFLD,^44^ breast cancer,^45^ lung cancer,^46^ and gastric cancer.^47^ We have established *CD36* transgenic fish overexpresses human *CD36* in the liver of zebrafish. *CD36* transgenic fish fed with high fat diet for 15 days significantly increased lipid accumulation, and extend high‐fat diet to 30 days induced HCC formation. Using *CD36* transgenic fish high‐fat diet model, we demonstrated that fucoidan exhibited a stronger anti‐lipid accumulation effect, as evidenced by Oil Red staining (Figure 3A) as well as LipidGreen analysis for liver (Figure 3B). In the *CD36* transgenic fish high‐fat diet for 30 days promoted HCC model, OF prevented the HCC formation (Figure 3C) and reduced PCNA protein markers expression revealed by IHC analysis (Figure 3D). Using qPCR to analyze the marker genes for lipogenesis (*srebf1*), cholesterol synthesis (*hmgcs1*), lipid oxidation (*acox3*), ER stress (*atf6*), inflammation (*il6*), and cell proliferation (*ccne1*), our results demonstrated OF significantly decreased the expression of all markers, especially *ccne1, srebf1, hmgcs1, and il6* (Figure 3E), indicating OF can prevent cell proliferation, lipid metabolism, and inflammation in *CD36* transgenic fish under high‐fat diet. Hence, OF exhibits strong effect on anti‐lipid accumulation and anti‐liver cancer formation in *CD36* transgenic zebrafish larvae model.

### 3.4 Microarray and pathways analysis identified important targets for OF effect

**FIGURE 4:**
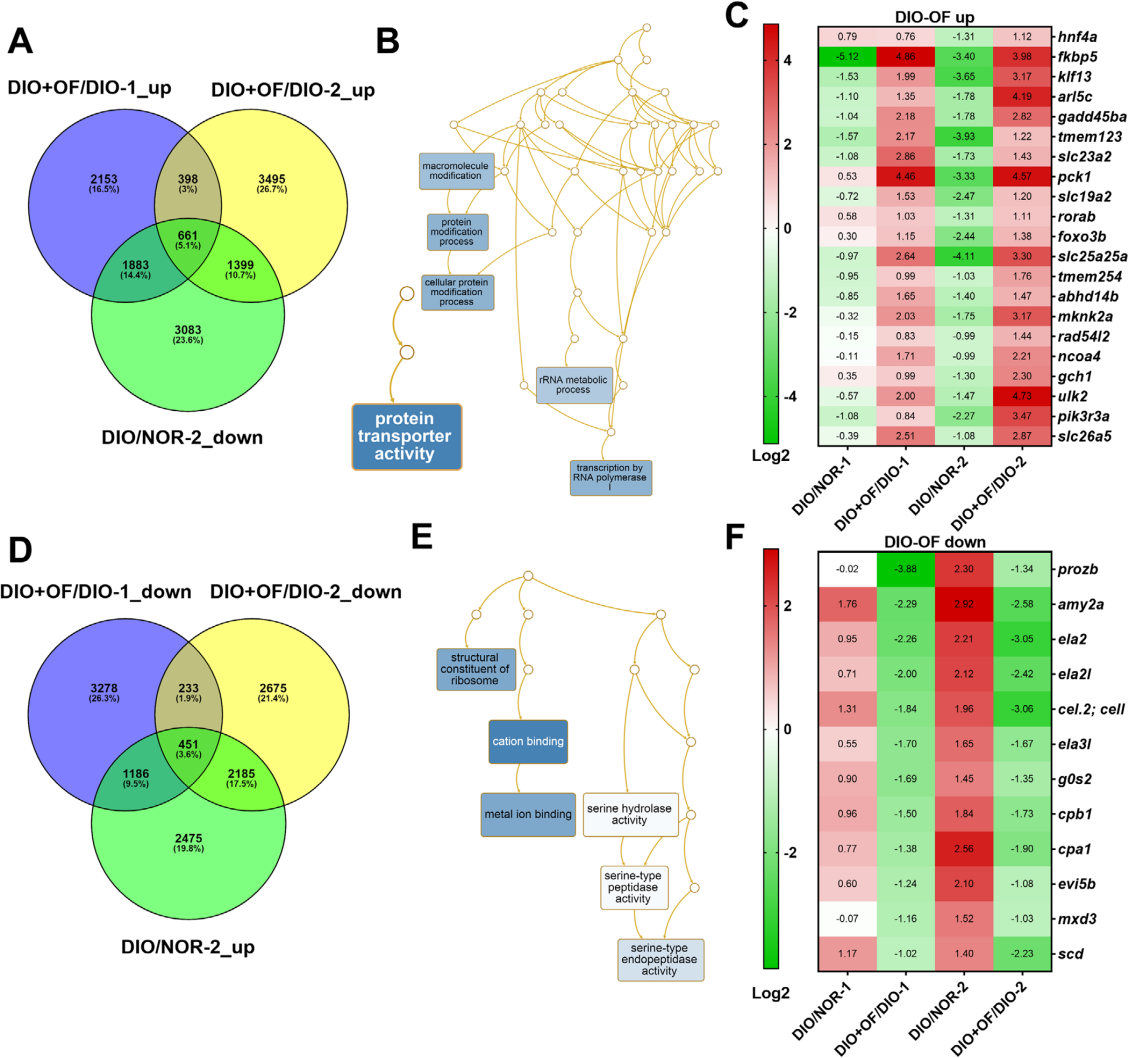
Pathways analysis for the differential expressed genes in [*HBx,src*], and [*HBx,src,p53^−/+^*] transgenic zebrafish following oligo‐fucoidan treatment. A, Venn diagram of genes was upregulated in oligo‐fucoidan pretreatment but downregulated by DIO and carcinogens. B, Network analysis of oligo‐fucoidan induced genes. C, The heatmap of top 21 oligo‐fucoidan upregulated genes. D, Venn diagram of genes was downregulated in oligo‐fucoidan pretreatment but up‐regulated by DIO and carcinogens. E, Network analysis of oligo‐fucoidan downregulated genes. F, The heatmap of top 12 oligo‐fucoidan downregulated genes.

To understand the molecular mechanisms underlying the OF inhibits hepatocarcinogenesis, we analyzed the whole‐genome expression profiles in order to explore OF‐mediated gene expression in liver. Total 661 candidate genes were downregulated by carcinogenesis (comparing DIO vs NOR in [*HBx,src,p53^−/+^*] transgenic fish) and reverted by OF treatment (comparing DIO+OF vs DIO in [*HBx,src*] and [*HBx,src,p53^−/+^*] transgenic fish) (Figure 4A). Using gene ontology analysis via WebGestalt,^48^ we found protein transporter and transcription by RNA polymerase I were enriched (Figure 4B, Figure S2A). The top upregulated genes were shown as heatmap where downregulated in DION/NOR and upregulated in DIO+OF/DIO in both [*HBx,src*] (#1) and [*HBx,src,p53^−/+^*](#2) (Figure 4C). Total 451 candidate genes were upregulated during carcinogenesis and reverted by OF treatment (Figure 4D), and those genes enriched in response to extracellular stimulus and cation binding and nonhomologous end‐joining (NHEJ) (Figure 4E, Figure S2B). The top downregulated genes were shown as heatmap where upregulated in DION/NOR and down‐regulated in DIO+OF/DIO in both [*HBx,src*] (#1) and [*HBx,src,p53^−/+^*](#2) (Figure 4F).

To find out the driver gene regulated by OF, we performed NetworkAnalyst, and identified 23 differential expressed genes were overlapping from four different groups of fish treated OF. Among them, hepatocyte nuclear factor 4A (*hnf4a*) is a driver gene,^31^ indicating *hnf4a* might plays a central role in various carcinogenesis insults in transgenic zebrafish.

Using Ingenuity Pathway Analysis (IPA) we find out other upstream regulators (Figure S3A), including *MYCN* (Figure S3B), *KRAS* (Figure S3C), *TGFB1* (Figure S3D), and *STK1* (Figure S3E), those genes were predicted to have enhanced expression in DIO versus NOR in [*HBx,src,p53^−/+^*] transgenic fish, and were repressed in DIO+OF compared to DIO. They upregulates (as shown in red) or downregulates the downstream target genes (as shown in green) in [*HBx,src,p53^−/+^*] transgenic fish.

**FIGURE 5:**
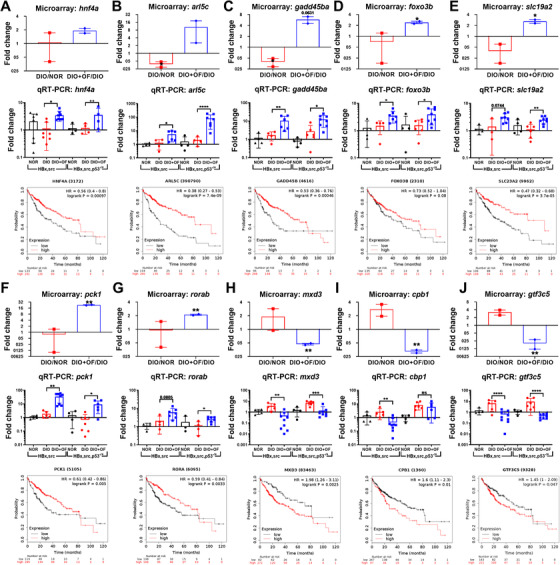
Validate the microarray data with qPCR for the five genes upregulated by OF (A‐G), and three genes downregulated by OF (H–J) in [*HBx,src*] and [*HBx,src,p53^−/+^*] transgenic zebrafish. The OF upregulated and downregulated genes from microarray and survival probability in liver cancer patients according to The Cancer Genome Atlas (TCGA) database. A‐G, Genes were upregulated by oligo‐fucoidan correlated to prolong survival rate in HCC patients. H‐J, Genes were downregulated by oligo‐fucoidan, and their expression correlated to poorer survival rate in HCC patients. Statistical significance was calculated by *t*‐test: *P ≤ .05, **P ≤ .01, ***P ≤ .001.

We further verify microarray data with qPCR by selecting top regulated genes to confirm the microarray results. Our qPCR results indicated that *hnf4a*, *arl5c*, *gadd45ba*, *foxo3b*, *slc19a2*, *pck1*, and *rorab* were upregulated by OF (Figure 5A–G), and *mxd3*, *cbp1* and *gtf3c5* were downregulated by OF (Figure 5H–J). According to The Cancer Genome Atlas database, OF upregulated genes are associated with better overall survival probability in patients with liver cancer (Figure 5A–G), however, those OF downregulated genes are associated with poor survival (Figure 5H–J). These data provide insights that OF might offer an anti‐HCC effect and prolong the survival.

### 3.5 Upregulation of hnf4a, asgr, and tdo2a by OF pretreatment in [HBx,src,p53^−/+^] transgenic zebrafish

**FIGURE 6:**
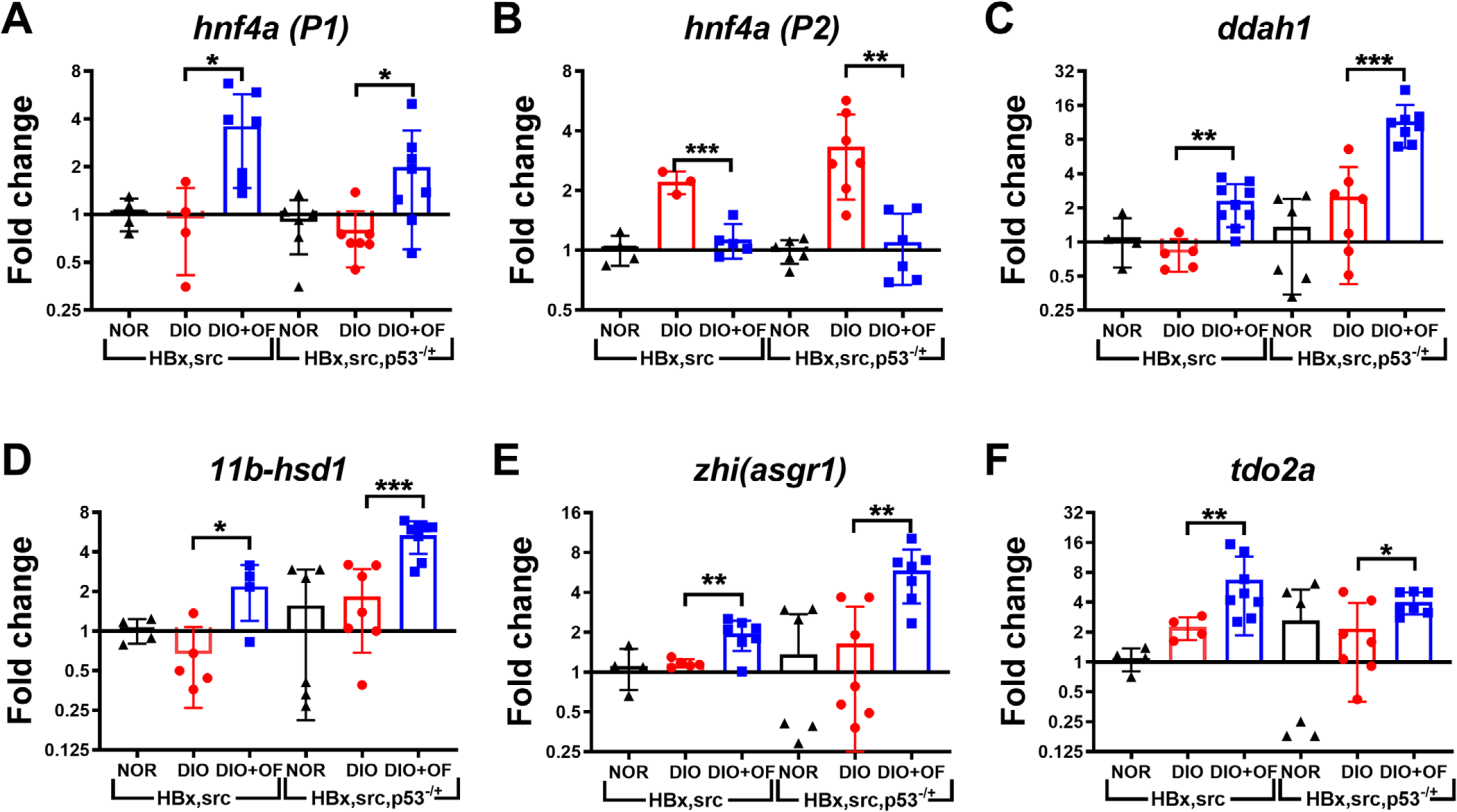
Upregulation of *hnf4a‐P1*, downregulation of *hnf4a‐P2*, and increased expression of *ddah1*, *11b‐hsd1, asgr1*, and *tdo2a* in zebrafish. Gene expression profiles of *hnf4a‐P1*, *hnf4a‐P2, ddah1*, 1*1b‐hsd1, zhi(asgr1)*, and *tdo2a*, in [*HBx,src*] and [*HBx,scr,p53^−/+^*] transgenic fish with normal diet (NOR), diet‐induced obesity promoting HCC (DIO) and oligo‐fucoidan treatment (DIO+OF) were shown. Statistical significance was calculated by *t*‐test: *P ≤ .05, **P ≤ .01, ***P ≤ .001.

We identified zebrafish *hnf4a* is the driver gene for those OF enriched genes from microarray, overexpression of mouse Hnf4a resets hepatocyte transcriptional network in and prevents hepatic failure.^49^ There are two isoforms of HNF4A, P1 isoform of HNF4A is tumor suppressor and P2 isoform of HNF4A acts as oncogene.^50^ Fucoidan has been demonstrated previously as a potential agonist for the C‐type lectin‐like receptor 2 (CLEC‐2) in platelets.^27^ We proposed OF binds to ASGR, the major CLEC‐2 receptor in hepatocytes,^28^ and upregulated P1‐isoform of HNF4A and downregulated the P2‐isoform of HNF4A. After qPCR analysis, we verified that OF pretreatment induced the expression of P1 isoform of *hnf4*a (Figure 6A) and reduced the P2 isoform of *hnf4a* (Figure 6B). We also confirmed that OF treatment induced the expression of dimethylarginine dimethylaminohydrolase 1 (*ddah1*) (Figure 6C), and 11Beta‐hydroxysteroid dehydrogenase type 1 (*11b‐hsd1*) (Figure 6D). *ddah1* and *11b‐hsd1* were found in the overlapping 23 genes in four groups of zebrafish treated by OF, they exhibited anti‐steatosis, anti‐inflammation and anti‐fibrosis effect.^31^ Furthermore, we found increased expression of ASGR orthologues*‐zhi*(*asgr1*) (Figure 6E) and *hnf4a* downstream target gene‐*tdo2a* expressions in both of the [*HBx,src*] and [*HBx,src,p53^−/+^*] fish with DIO (Figure 5D). OF treatment in transgenic zebrafish induced expression of P1‐*hnf4a*, *asgr1*, and *hnf4a* downstream target genes which might provide a valuable hint for the multiple bio‐function of OF.

### 3.6 OF binds to C‐type lectin‐like receptor 2 (CLEC‐2)‐ASGR1/2 in hepatoma cells, enhances pSTAT3, which binds to P1‐HNF4A promoter in hepatoma cells

**FIGURE 7:**
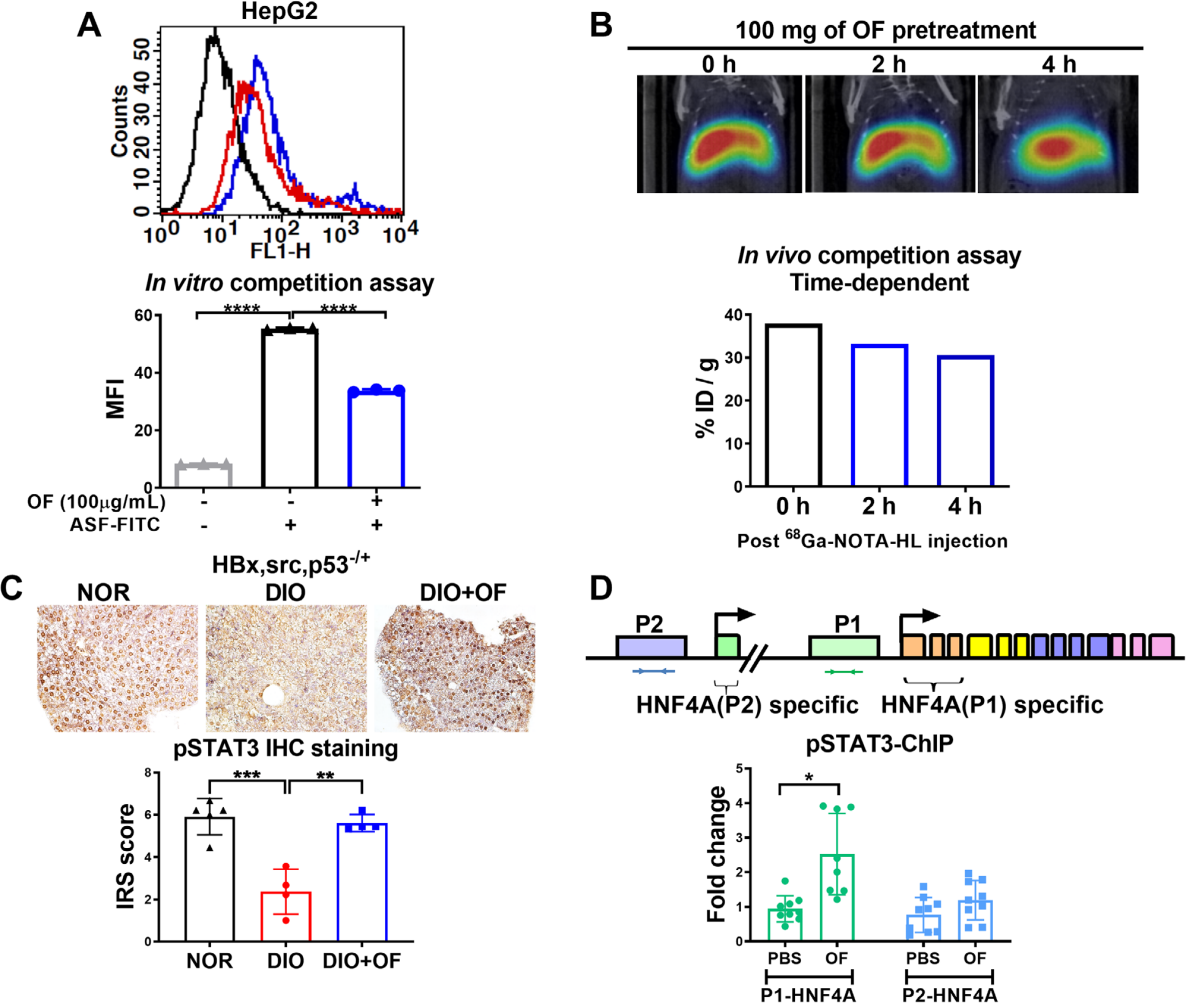
Oligo‐fucoidan binds to ASGR1/2 in hepatoma cells, enhances pSTAT3 in hepatic tissues of [*HBx,src,p53^−/+^*] transgenic fish, and enriches pSTAT3 binding to P1‐HNF4A promoter in hepatoma cells. A, Flow cytometry profiles of HepG2 cells. The mean fluorescence intensity (MFI) profiles of HepG2 cells with OF treatment. The asialofetuin‐FITC (ASF‐FITC) signals were presented in the control (black), without OF co‐treatment (blue) and with OF co‐treatment. B, The reduction of ^68^Ga‐NOTA‐HL radioactivity intensity in a time‐dependent manner was depended on the administration of OF (N = 1). Relative radioactive intensity of ^68^Ga‐NOTA‐HL in vivo between different time courses. Black bar indicates post‐^68^Ga‐NOTA‐HL injection 0 hour, blue and red bar denote post‐^68^Ga‐NOTA‐HL injection 2 hours and 4 hours, respectively. C, Immunohistochemistry staining of pSTAT3 in [*HBx,src,p53^−/+^*] transgenic fish fed with normal diet (NOR), diet‐induced obesity (DIO) or treated with oligo‐fucoidan (DIO+OF). Semi‐quantitative analysis of pSTAT3 staining in hepatic tissues from [*HBx,src,p53^−/+^*] transgenic zebrafish. Statistical significance was calculated by t‐test: **P ≤ .01, ***P ≤ .001. D, The expression fold of chromatin immunoprecipitation (ChIP) of pSTAT3 binding to P1‐HNF4A promoter in HepG2 cells was enriched by OF treatment. Statistical significance was calculated by *t*‐test: *P ≤ .05, **P < .01, ***P ≤ .001; **** *P* ≤ .0001.

To verify OF binds to C‐type lectin‐like receptor 2‐ASGR on hepatoma cells, we used HepG2 hepatoma cell treated with OF using in vitro competition assay. Veritably, OF reduced the binding capacity of FITC‐labeled asialofetuin, an ASGR‐binding protein within hepatocyte (Figure 7A). We have performed in vivo competition assay in murine, ^68^Ga‐NOTA‐HL (galactose binding to ASGR) was injected together with OF via tail vein injection, and found OF reduced the radioactivity of ^68^Ga‐NOTA‐HL than PBS with statistically significance.^30^ Here, using time‐dependent assay for in vivo competition assay, we observed attenuation of ^68^Ga‐NOTA‐HL radioactivity in a time‐dependent manner after OF pretreatment (Figure 7B). These data suggest that OF specifically binds to hepatic ASGR in vivo and in vitro.

The ASGR activates JAK2‐STAT3 signaling pathways for desialylated platelets induced by inflammatory responses.^29^ We hypothesized that after binding to ASGR, OF activates STAT3 phosphorlyation and enhances the nuclearization of pSTAT3, which binds to the promoter of *HNF4A* and increases *HNF4A* mRNA expression. In [*HBx,src,p53^−^* ^/+^] transgenic fish with DIO, oral feeding with OF increased pSTAT3 expression (Figure 7C). Statistical analysis revealed that the significant reduction of pSTAT3 expression after DIO was rescued by OF pretreatment in [*HBx,src,p53^−/+^*] transgenic fish with DIO (Figure 7C).

There are two isoforms of HNF4A, P1 isoform of HNF4A is tumor suppressor and P2 isoform of HNF4A acts as oncogene.^50^ OF pretreatment induced the expression of P1 isoform of *hnf4a* in transgenic zebrafish. To investigate whether OF promotes pSTAT3 binding to *HNF4A* promoter, ChIP was performed using HepG2 hepatoma cell. After adjusted to IgG controls and then scored with the pSTAT3 antibody, we found OF pretreatment specifically enriched pSTAT3 binding to *P1‐HNF4A* promoter (Figure 7C). This novel findings highlight the mechanism underlines the OF activates the pSTAT3 and then increases the expression level of P1‐HNF4A via pSTAT3 binding to the promoter of P1‐HNF4A.

### 3.7 OF inhibits the hepatoma cell proliferation via ASGR/STAT3/HNF4A pathway

**FIGURE 8:**
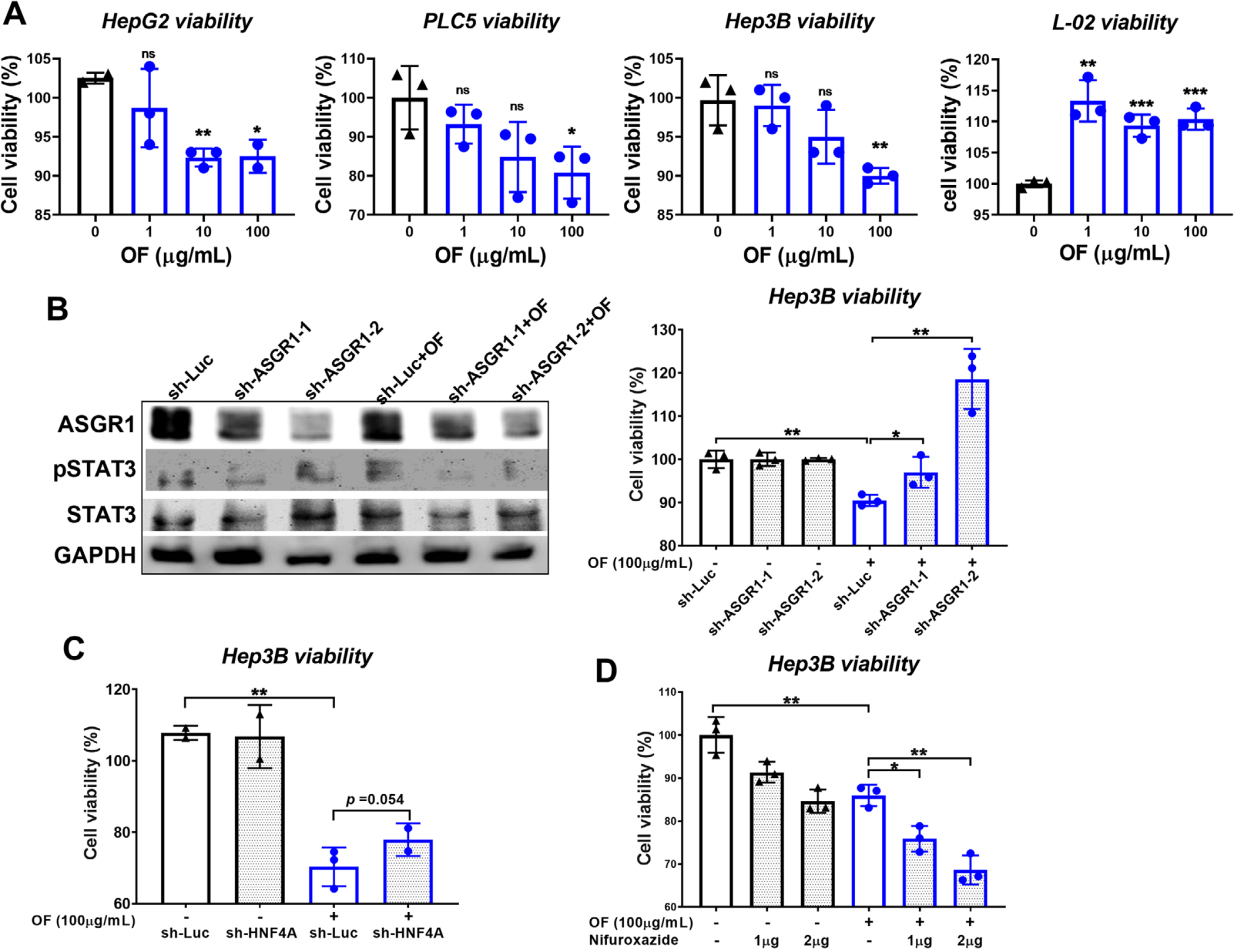
Knockdown of ASGR and HNF4A reverses the effect of oligo‐fucoidan reduced the hepatoma cell viability. A, Oligo‐fucoidan reduced the cell viability of three hepatoma cells. B, Western blot analysis indicating that knockdown of ASGR by sh‐ASGR1‐1 and sh‐ASGR1‐2 reduced the ASGR protein level and pSTAT3. Cell viability assay demonstrated that OF treated for 48 hours significantly reduced cell proliferation and sh‐ASGR1‐1 and sh‐ASGR1‐2 significantly reversed the OF effect. C, Knockdown of HNF4A by sh‐HNF4A reverse the OF inhibiting hepatoma cell viability. D, STAT3 inhibitor synergize with oligo‐fucoidan on reduction of hepatoma cell viability.

We treated fucoidan to three different hepatoma cells and found the cellular viability was decreased in a dosage dependent manner (Figure 8A). On the contrary, OF enhanced the normal liver cellular viability in L‐02 cell (Figure 8A). These data affirm the specific effect of OF on hepatoma cells and normal hepatocytes. Thus, OF is a potential anti‐liver cancer and liver‐protective agent.

To explore whether OF anti‐HCC effect is through the ASGR/STAT3/HNF4A pathway, we knockdown the ASGR and HNF4A in Hep3B hepatoma cells, and performed the functional analysis for cell viability assay. Knockdown of ASGR1 with shRNA decreased ASGR1 protein levels, and sh‐ASGR1‐2 had stronger inhibition than sh‐ASGR1‐1 (Figure 8B). As shown in western blot, increase of pSTAT3 by OF was reversed by knockdown of ASGR1 (Figure 8B). OF decreased the hepatoma cell viability (in sh‐Luc), and sh‐ASGR1 reversed the effect of OF (Figure 8B). Similarly, knockdown HNF4A reversed the effect of OF (Figure 8C). STAT3 is a potential drug target for cancer therapy, and STAT3 phosphorylation inhibitor, Nifuroxazide can decrease the viability of multiple cancer. Even though we found OF can increase the pSTAT3, we wonder whether STAT3 inhibitor has anti‐HCC effect together with OF. Surprisingly, there is synergistic effect between Nifuroxazide and OF on reducing the hepatoma cell viability (Figure 8D). Since OF regulates many pathways, it could be very possible that OF acts through other pathways synergize with STAT3 inhibitor to have stronger anti‐HCC effect. Nevertheless, our results indicate that OF has synergistic effect with other combinatorial therapeutics against cancers.

## 4 DISCUSSION

Fucoidan exhibits anti‐obesity, anti‐inflammatory, and tumor‐retardation effects.^5^, ^20^, ^51^, ^52^, ^53^ We used low molecular weight (500‐1500 Da) OF in this study, and the novel finding is that OF reduced HCC formation in three different models: [*HBx*,*src*] and [*HBx*,*src,p53^−/+^*] diet‐induced obesity, and *CD36* high‐fat diet transgenic fish models. In addition to our previous finding that OF prevents radiation induced fibrosis and secondary tumor,^31^ we demonstrated OF exhibits the anti‐HCC, anti‐steatosis, and anti‐liver fibrosis effect in zebrafish models from many different insults. We also demonstrated that OF binds to ASGR1, activating pSTAT3 which specifically binds to P1‐HNF4A promoter in HepG2 hepatoma cells. Knockdown of ASGR1 or HNF4A reverse the OF mediated hepatoma cell proliferation inhibition effect. Those results echo our previous finding that OF binds to ASGR, increases P1‐HNF4A through activating pSTAT3 on normal hepatocyte.^30^ HNF4A plays crucial role in liver inflammatory networks,^54^ suppresses liver cancer formation^55^ and activation. HNF4A by small activating RNA could prevent NAFLD and improve metabolic profile.^56^ In this study, we reveal OF treatment could induce the expression of P1‐HNF4A through activating pSTAT3 binding to the P1‐promoter, this discovery may emphasize the therapeutic roles of OF on anti‐fatty liver, anti‐fibrosis, anti‐HCC in different genetic, and diet insults.

Recently, fucoidan has been reported as a new, natural anti‐obesity agent that reduces fat absorption and inhibits adipose‐related gene expression.^57^, ^58^ Moreover, it also plays a role in reducing chemotherapy side effects, such as inflammation.^59^ We have demonstrated that OF increased the immune system and anti‐viral protein expression, and decreased the expression of lipid accumulation, fibrosis, cancer markers in wild‐type zebrafish.^30^ We also proved OF prevents radiation induced fibrosis and secondary tumor formation.^31^ Previously, OF inhibits radiation induced lung fibrosis in mouse model through reducing inflammatory cytokine,^53^ and fucoidan inhibits cell proliferation, migration, and cell arrest in HepG2 hepatoma cell.^60^ In this study, we further demonstrated that OF effectively reducing HCC incidence in different transgenic zebrafish HCC models including diet‐induced obesity of [*HBx,src*] and [*HBx,src,p53^−/+^*] models, as well as *CD36* transgenic zebrafish under high‐fat diet induced HCC. We uncovered the potential mechanism of fucoidan for inhibition hepatoma cell proliferation, but there is still a need for further examination of OF dosages in real practice on humans, because various sources and dosages have been used.^5^

There is much clinical and scientific interest in searching among natural products additional active ingredients that could help develop new nature‐based drugs and assist conventional and herbal drugs currently available for treating human diseases.^61^ Previous and current nature‐based drugs were derived preferentially from plants including fungi, in addition to bacteria, and rarely obtained from animals or maritime sources like algae. Our material in the study is from the marine brown seaweeds and potentially inhibits irradiation‐related liver fibrosis and risk of HCC.^31^ Till now, there is no study to show drugs or natural products could inhibit liver fibrosis and risk of HCC. Our study is the first study to show the outcomes to inhibit liver fibrosis and risk of HCC. The potentially anti‐HCC effect of low molecular weight fucoidan in diet‐induced‐obesity [*HBx,src*], [*HBx,src,p53^−/+^*], and *CD36‐*related HCC might apply to many different risk factors of HCC. However, low molecular weight fucoidan will only be accepted by consumers and regulatory agencies if efficacy for certain well defined diseases like [*HBx,src*], [*HBx,src,p53^−/+^*], and *CD36‐*related HCC has been established using randomized controlled trials, and if severe adverse effects had not been observed, providing thereby a favorable profile of benefits over risks.^61^ Currently, A randomized, double‐blind, controlled trial was conducted evaluating the efficacy of OF in advanced HCC patient (ClinicalTrials.gov Identifier: NCT04066660) in Shanghai Zhongshan Hospital by Hi‐Q Marine Biotech International, Ltd., hopefully we could uncover the results in human patients soon.

Fucoidan is complex sulfated polysaccharide derived from brown seaweed, usually high molecular weight. In this study, the fucoidan was further hydrolyzed with a glycolytic enzyme to low molecular weight with average 1200 Da,^34^ and contains 234.48 ± 0.08 μmol/g fucose and 49.14 ± 0.07 μmol/g galactose.^32^ High molecular weight fucoidan can bind to C‐type lectin‐like receptor 2 (CLEC‐2), induce receptor clustering and platelet activation via tyrosine kinase‐dependent pathway.^27^ Low molecular weight fucoidan may bind to CLEC‐2 without inducing the receptor clustering, and here we showed OF can bind to CLEC‐2 receptor on hepatocyte, that is ASGR.^62^ The structure of OF might provide some hints about its association with CLEC‐2. Although, the heterogeneity of fucoidan makes difficult to complete elucidate its structure, fucoidan mainly contains of fucose and galactose. Low molecular weight fucoidan contains 35% of galactose with more potent endothelial protection activity was reported.^63^ Based on the primary structure and conformation in aqueous solution, fucoidan from different species contains sulfated galactose or sulfated fuco‐galactose side chains, and those branches play significant role in the intestinal immunological activity.^64^ From screening of 320 oligosaccharides array, C‐type lectin‐like receptor CLEC4C specifically recognizes complex type sugars with terminal residues of β1–4‐ or β1–3‐galactose.^65^ Those results indicated galactose side chain is the active ingredient of OF bind to ASGR, the CLEC‐2 receptor on hepatocyte.

The anti‐cancer activities of OF have been demonstrated in many cancers and have been as an adjuvant with clinical drugs. Fucoidan mediated multiple pathways including NF‐κB, PI3K/Akt and MAPK, ER stress, ROS, to induce anti‐proliferation effect.^66^ Fucoidan as an adjuvant combined with FOLFOX for the advanced colorectal cancer patients and fucoidan combined with tamoxifen, cisplatin, and paclitaxel enhanced the anti‐cancer effect on breast cancer. Fucoidan has synergistic effect when combined with tyrosine kinase inhibitor lapatinib on esophageal carcinoma cell line which has been reported.^66^ In this study, we found pSTAT3 can be activated by OF, however, when we combined the STAT3 inhibitor with OF, we observed synergistic effect on inhibiting hepatoma cell proliferation. This phenomenon can be explained by the fucoidan mediated activation of multiple pathways. Nevertheless, the detail mechanism requires more investigation.

We demonstrated the anti‐HCC effect of OF on different transgenic fish models with [*HBx,src*], [*HBx,src,p53^−/+^*], and *CD36‐*related HCC. We propose OF can prevent liver damage by reset the genetic regulatory network through upregulates HNF4A‐P1 isoform. Our data strongly support the beneficial effects of OF with regard to the inhibition of liver fibrosis and lipogenic enzyme formation, preventing the progression of consequent hepatocarcinogenesis. The current study demonstrates OF is an effective therapeutic agent not only for zebrafish animal models, but also in hepatoma ASGR‐expressed HepG2, PLC5 and Hep3B cells. OF activates phosphorylation of STAT3 and enhances the binding to P1‐promoter of HNF4A which is the potential mechanism for reducing hepatocarcinogenesis in liver cancer. Moreover, OF prevents radiation induced fibrosis and secondary tumors,^31^ also exhibits protection effect in normal hepatocyte.^30^ Furthermore, OF is a safe food supplement without toxicity at the dose of 2000 mg/kg body weight/day was used in mouse, so the OF could be an excellent therapeutics for liver cancer patients.

## 5 CONCLUSIONS

We proved that OF binds to ASGR1/2 receptors on hepatoma cell, we also provided strong evidence that OF activates phosphorylation of STAT3, and then pSTAT3 will bind to P1 promoter of HNF4A, transcriptionally regulates the level of HNF4A, P1 isoform. Our study provides an insight into the mechanism for OF applied to liver diseases. We suggest that OF not only prevents many liver diseases and liver cancer caused by genetic and dietary factors, moreover, OF exhibits both anti‐hepatocarcinogenesis and hepatoprotection effect, which is the most challenging part for many therapies using small‐molecule drugs in clinical practice.

## CONFLICT OF INTEREST

The authors declare that there is no conflict of interest that could be perceived as prejudicing the impartiality of the research reported.

## AUTHOR CONTRIBUTIONS

Drafting of the manuscript: Yuh. Obtained funding: Yuh. Study supervision: Yuh. Project administration: Yuh. Methodology: Szu‐Yuan Wu. Performed the experiments, validation, and investigation: Yang, Cheng, Sampurna, Chan, and Lin. All authors have read and agreed to the current version of manuscript.

## Supporting information

Figure S1

Supporting Information

## Data Availability

The data used in the current study are available from the corresponding author on request. The raw data of the microarray have been submitted to the NCBI Gene Expression Omnibus (GEO) under accession code GSE148501.
