# Supplementary material for: Low molecular weight fucoidan inhibits hepatocarcinogenesis and nonalcoholic fatty liver disease in zebrafish via ASGR/STAT3/HNF4A signaling
Source: Clin Transl Med. 2017 Jul 28;10(8):e252. doi: 10.1002/ctm2.252 (PMC7752165; doi:10.1002/ctm2.252)
Supplement: Supplementary file 1 — Figure S1 [file CTM2-10-e252-s001.pdf]

FIGURE S1

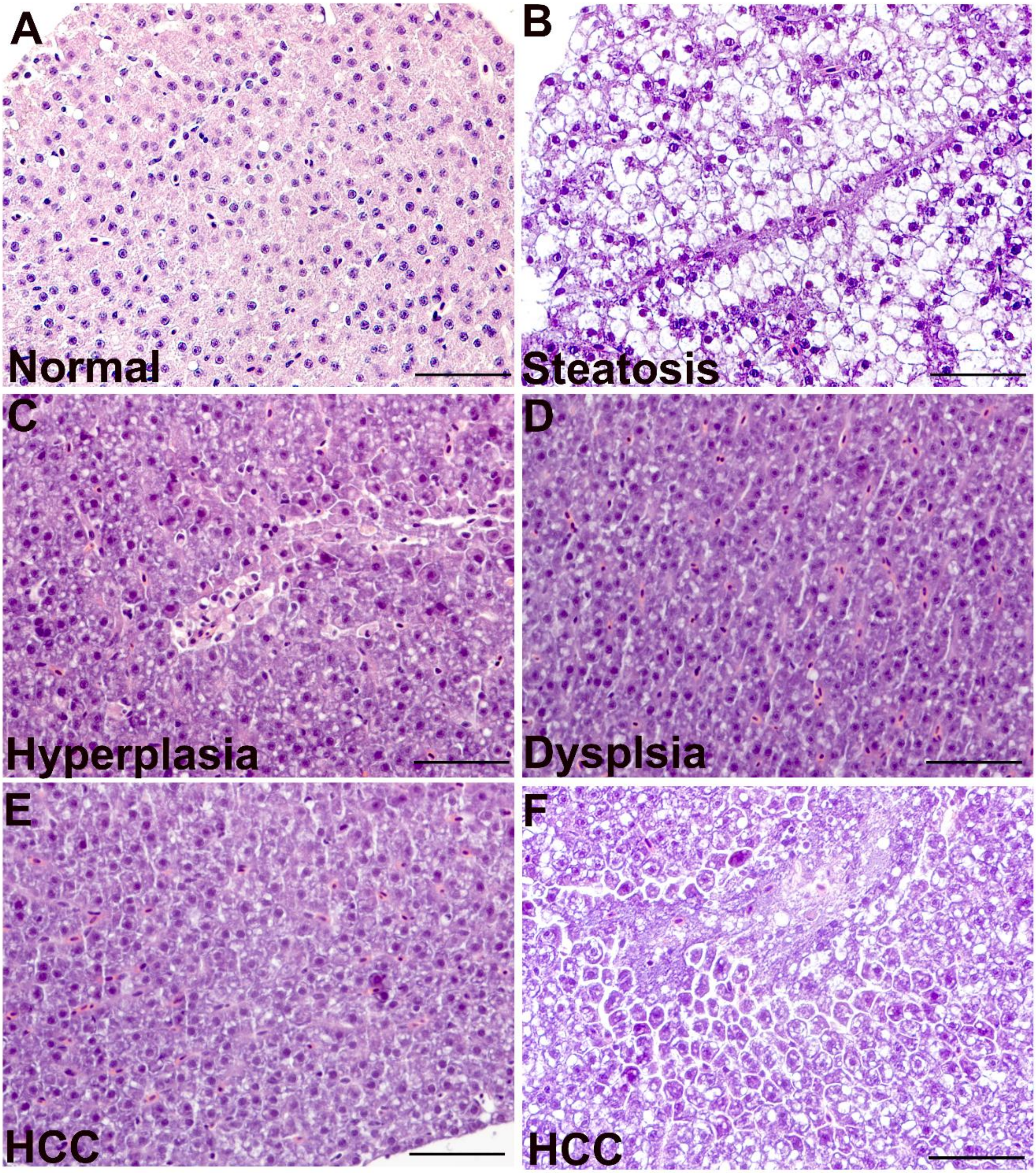

Figure S2

A DIO+OF/DIO upregulated genes enriched pathways

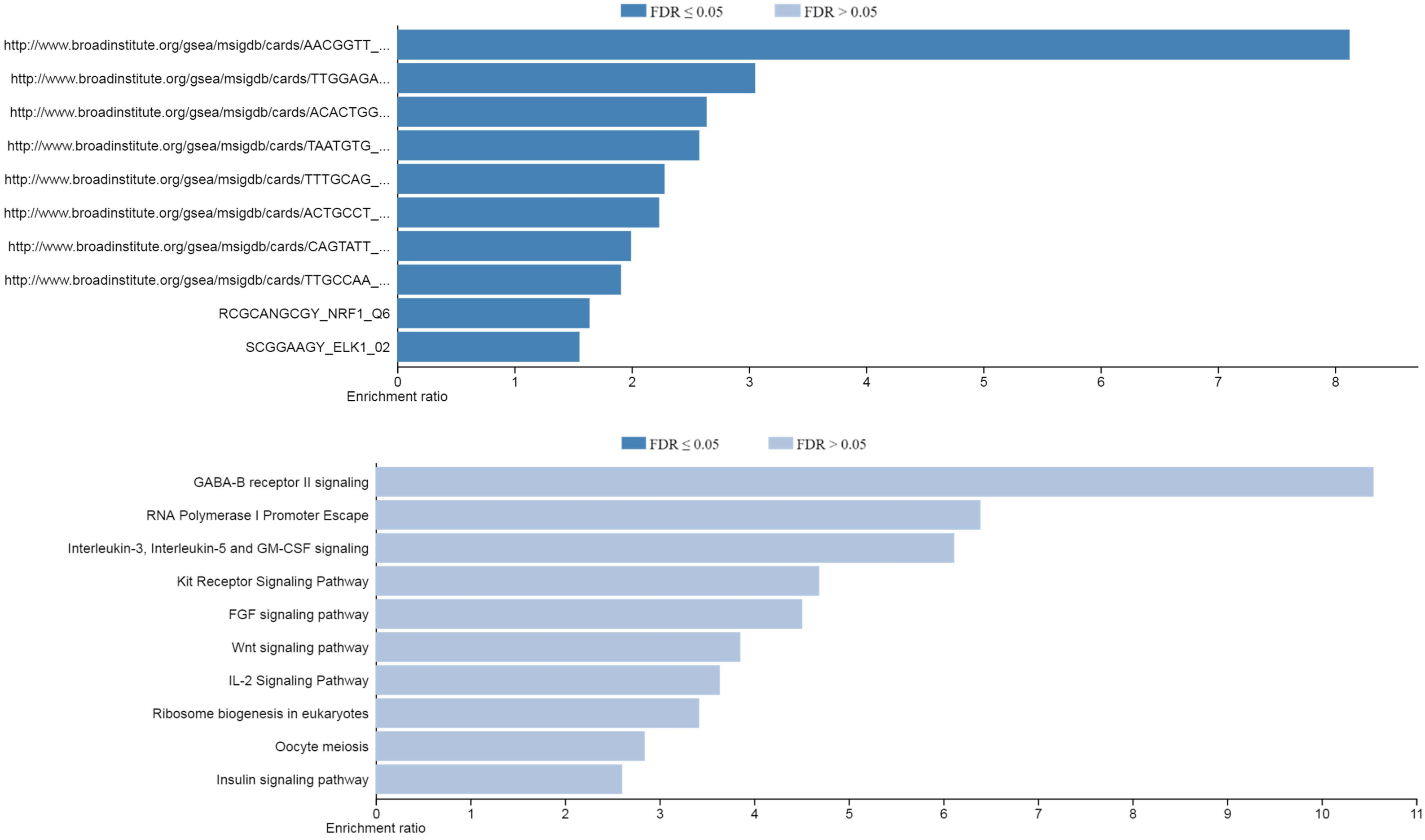

B DIO+OF/DIO downregulated genes enriched pathways

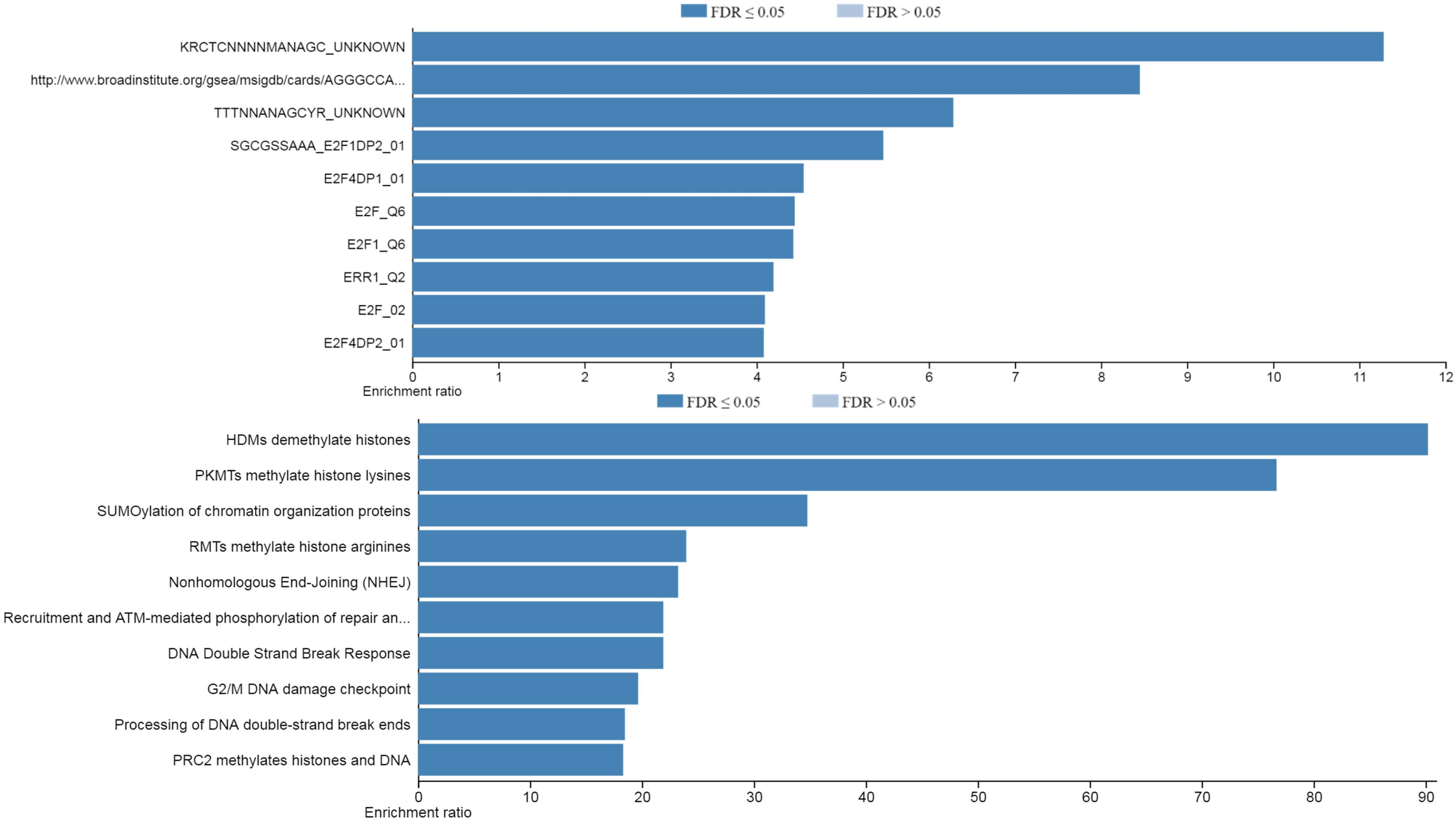

Figure S3

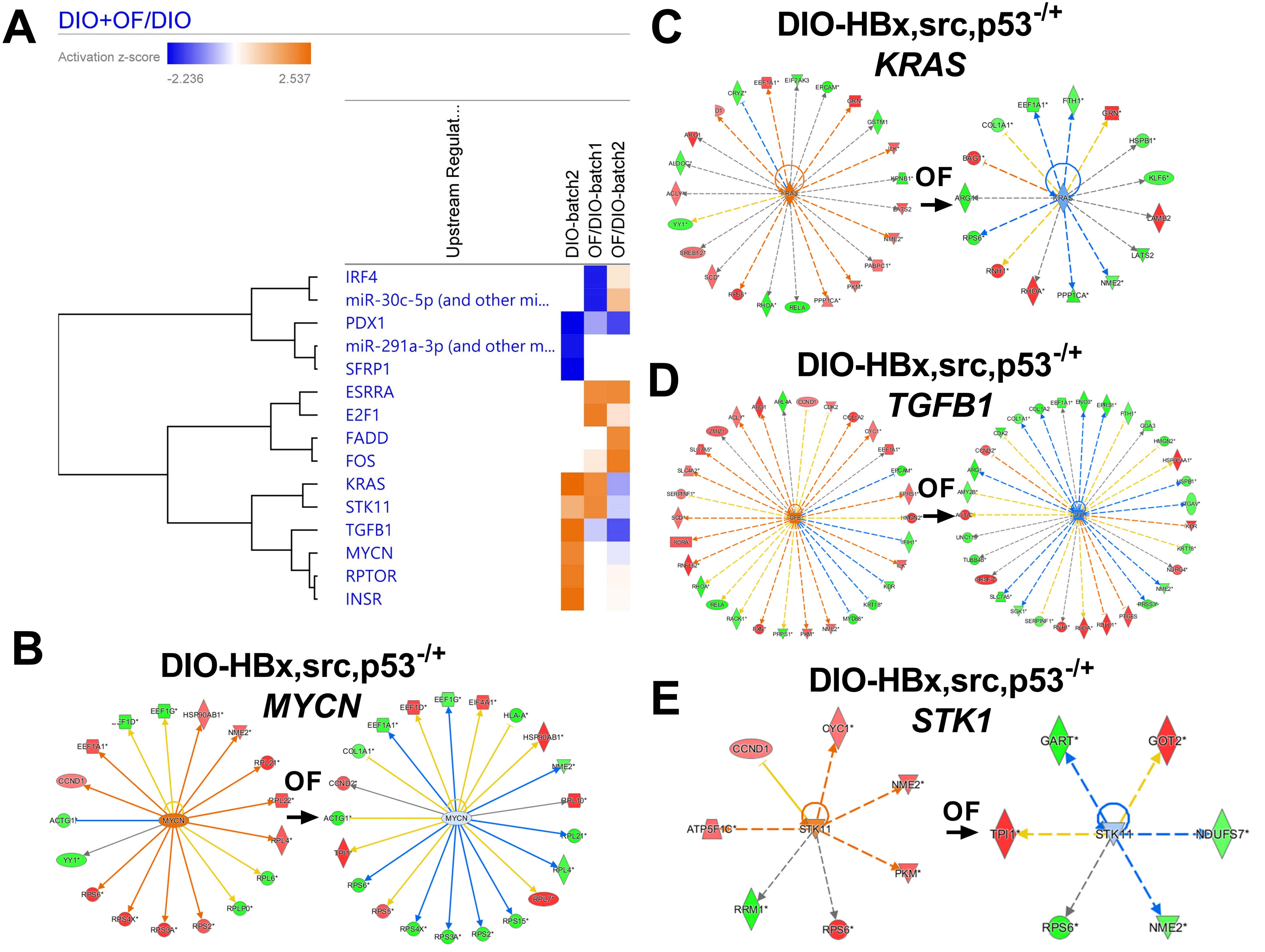
A
DIO+OF/DIO
Activation z-score
-2.236
2.537
Upstream Regulat...
DIO-batch2
OF/DIO-batch1
OF/DIO-batch2
IRF4
miR-30c-5p (and other mi...
PDX1
miR-291a-3p (and other m...
SFRP1
ESRRA
E2F1
FADD
FOS
KRAS
STK11
TGFB1
MYCN
RPTOR
INSR
B
DIO-HBx,src,p53-/+
MYCN
OF
C
DIO-HBx,src,p53-/+
KRAS
OF
D
DIO-HBx,src,p53-/+
TGFB1
OF
E
DIO-HBx,src,p53-/+
STK1
OF
CCND1
CYC1*
NME2*
ATP5F1C*
STK11
PKM*
RRM1*
RPS6*
GART*
GOT2*
TPI1*
NDUFS7*
