# Supplementary material for: Low molecular weight fucoidan inhibits hepatocarcinogenesis and nonalcoholic fatty liver disease in zebrafish via ASGR/STAT3/HNF4A signaling
Source: Clin Transl Med. 2017 Jul 28;10(8):e252. doi: 10.1002/ctm2.252 (PMC7752165; doi:10.1002/ctm2.252)
Supplement: Supplementary file 2 — Supporting Information [file CTM2-10-e252-s002.pdf]

Supplementary Materials:

Fig. S1. Representative images for (**A**) normal hepatocyte, (**B**) steatosis, (**C**) Hyperplasia, (**D**) Dysplasia, and (**E**,**F**) HCC in transgenic zebrafish.

Fig. S2. Pathways analysis for the differential expressed genes in [*HBx,src*], and [*HBx,src,p53*$^{-/+}$] transgenic zebrafish following oligo-fucoidan treatment. (**A**) Enriched pathways of oligo-fucoidan upregulated genes. (**B**) Enriched pathways of oligo-fucoidan downregulated genes..

Fig. S3. Upstream regulator analysis using IPA for the differential expressed genes in [*HBx,src*], and [*HBx,src,p53*$^{-/+}$] transgenic zebrafish following oligo-fucoidan treatment. (**A**) Clustering of the upstream regulators identified by IPA for the [*HBx,src,p53*$^{-/+}$]-DIO transgenic fish compare to NOR (DIO-batch2), [*HBx,src*]-DIO+OF compare to DIO (DIO-batch1), and [*HBx,src,p53*$^{-/+}$]-DIO+OF compare to DIO (DIO-batch2). (**B-E**) The upstream regulators predicted by IPA including *MYCN*, *KRAS*, TGFB1 and STK1 which were predicted induced by DIO (as shown in orange), and repressed by OF (as shown in blue) together they either upregulates the downstream genes (as shown in red) or inhibit the downstream genes (as shown in green) in [*HBx,src,p53*$^{-/+}$] transgenic fish.
